# Transient lipid-bound states of spike protein heptad repeats provide insights into SARS-CoV-2 membrane fusion

**DOI:** 10.1126/sciadv.abk2226

**Published:** 2021-10-08

**Authors:** Sai Chaitanya Chiliveri, John M. Louis, Rodolfo Ghirlando, Ad Bax

**Affiliations:** 1 Laboratory of Chemical Physics, National Institute of Diabetes and Digestive and Kidney Diseases, National Institutes of Health, Bethesda, MD 20892, USA.; 2 Laboratory of Molecular Biology, National Institute of Diabetes and Digestive and Kidney Diseases, National Institutes of Health, Bethesda, MD 20892, USA.

## Abstract

Entry of SARS-CoV-2 into a host cell is mediated by spike, a class I viral fusion protein responsible for merging the viral and host cell membranes. Recent studies have revealed atomic-resolution models for both the postfusion 6-helix bundle (6HB) and the prefusion state of spike. However, a mechanistic understanding of the molecular basis for the intervening structural transition, important for the design of fusion inhibitors, has remained elusive. Using nuclear magnetic resonance spectroscopy and other biophysical methods, we demonstrate the presence of α-helical, membrane-bound, intermediate states of spike’s heptad repeat (HR1 and HR2) domains that are embedded at the lipid-water interface while in a slow dynamic equilibrium with the postfusion 6HB state. These results support a model where the HR domains lower the large energy barrier associated with membrane fusion by destabilizing the host and viral membranes, while 6HB formation actively drives their fusion by forcing physical proximity.

## Teaser

Heptad repeat regions of SARS-CoV-2 spike protein transiently disrupt membrane integrity while driving cellular entry.

## INTRODUCTION

Enveloped severe acute respiratory syndrome coronavirus 2 (SARS-CoV-2), the causative agent of the coronavirus disease 2019 (COVID-19) pandemic, initiates infection by promoting its entry into the host cell. This viral entry is orchestrated by its spike (S) protein, which fuses the viral and host cell membranes. Because of its antigenic properties, the S protein also serves as a valuable target for vaccine development and all current vaccines target this protein. S protein belongs to the family of class I viral fusion proteins that have been studied extensively for many decades, with hemagglutinin of influenza and gp160 of HIV-1 explored the most (*1*). The homotrimeric S protein consists of two covalently linked subunits, S1 and S2. Cellular entry involves two distinct steps: binding to the host cell receptor, angiotensin-converting enzyme-2 (ACE2) through its S1 subunit, and subsequent fusion of the host and viral cell membranes, which is mediated by its S2 subunit (*2*).

Similar to other class I fusion proteins (*1*), for a successful fusion event, the S protein must undergo a cascade of conformational changes while transitioning from its prefusion to the postfusion state (*3*). X-ray and cryo–electron microscopy studies have reported a plethora of pre- and postfusion structures of multiple class I fusion proteins, providing important insights into the membrane fusion mechanism (*1*, *3*–*5*). In the prefusion state, ACE2 recognition followed by protease cleavage at S1/S2 and S2′ sites leads to dissociation of the S1 subunit, resulting in the formation of an extended prehairpin intermediate (PHI) conformation of S2 (*2*, *3*, *6*). In this conformation, the hydrophobic fusion peptide (FP) anchors into the host cell membrane; a heptad repeat domain-1 (HR1) adopts a three-helix bundle (3HB) arrangement, while the distal transmembrane (TM) domain is anchored to the viral membrane. In a series of structurally less-characterized events, another ectodomain helix, HR2, folds in an antiparallel direction onto HR1, resulting in a juxtaposed arrangement of FP and TM regions. Completion of the structural rearrangement into the formation of 6HB, often referred as the postfusion state, only takes place during or just after the pore formation (*7*). 6HB adopts a C_3_ symmetric structure, where HR1 forms an internal trimeric coiled-coil structure, stabilized by interhelical hydrophobic interactions at positions “a” and “d” of the helical wheel, whereas the hydrophobic residues at positions “e” and “g” of HR1 are sequestered by the exterior HR2 helices (*5*, *8*). The energy released by the folding events involved in the formation of 6HB is generally considered to play a key role in traversing the high-energy barrier required for the fusion of two hydrophobic, negatively charged membranes.

Intermediate states of membrane fusion have been targeted to restrict the transmission of enveloped viruses (*5*, *9*). Lipid-conjugated peptides (lipopeptides) demonstrated enhanced antiviral potency against several viruses, including SARS-CoV-2 (*10*, *11*). These inhibitors containing the HR2 peptide sequence bind to the HR1 region in the extended-PHI state, thereby stalling the formation of the postfusion state. While electron tomography studies provided low-resolution images of the extended-PHI state from other class I fusion proteins, high-resolution structures have not yet been determined (*12*). Most fusion models display FP and TM domains as the membrane-embedded regions of these fusion proteins, with heptad repeats (HR1 and HR2) generally illustrated as linear extended trimers in these intermediate states (*1*). However, following an initial report by Shin and coworkers on hemagglutinin (*13*), growing evidence indicates that HR1 and HR2 regions of the HIV-1 gp41 can interact with membranes and actively participate in the fusion process (*14*, *15*).

Our earlier study of a 6HB mimic of the HIV-1 gp41 ectodomain demonstrated that it dissociates into monomeric α-helices in the presence of fos-choline [*n*-dodecylphosphocholine (DPC)] micelles and interacts weakly with small unilamellar vesicles (SUVs) (*16*). Nuclear magnetic resonance (NMR) measurements also revealed the absence of HR1-HR2 interactions in the micelle-bound state and showed that these helices partition at the lipid-water interface (*16*). A recent solid-state NMR study of the gp41 HR1 and HR2 helices using uniaxially aligned POPC (1-palmitoyl-2-oleoyl-glycero-3-phosphocholine) and POPC/POPG [sodium salt of 1-palmitoyl-2-oleoyl-*sn*-glycero-3-phospho-(1′-rac-glycerol)] bilayers reached the same conclusion and found that these helices are parallel to the membrane surface (*17*). Thus, both solution and solid-state NMR studies support an intermediate membrane-bound state where the HR2 and HR1 helices are embedded at the lipid-water interface of the viral and host cell membranes, respectively. In an analogous homotypic membrane fusion pathway, the HR1 domain of the mitochondrial membrane protein, Mitofusin, was shown to mediate membrane fusion by anchoring in the lipid bilayer and thereby priming it for fusion (*18*).

To evaluate whether the membrane-bound intermediate stage is an obligatory step in the membrane fusion process mediated by class I fusion proteins, we carried out biophysical measurements that characterize the membrane binding properties of both HR1 and HR2 regions of the SARS-CoV-2 spike (or fusion) protein. The isolated water-soluble HR1 and HR2 domains were found to exist in a monomer-tetramer equilibrium, consistent with tetrameric self-association property of these peptides derived from SARS-CoV and HIV-1 viruses (*19*, *20*). In the presence of phospholipid isotropic bicelles or SUVs, we find that these HR regions adopt monomeric α-helical structures, as evidenced by NMR spectroscopy, circular dichroism (CD), and analytical ultracentrifugation (AUC) measurements. Both hydrogen exchange (HX) and ^15^N backbone relaxation measurements point to a dynamic HR1 α-helix with large internal motions on a subnanosecond time scale. A solution structure derived from residual dipolar couplings (RDCs) indicates that HR1 includes a dynamic kink in its helical structure, whereas paramagnetic relaxation identifies the residues that are exposed to solvent and to the phospholipid bilayer. Addition of the HR2 peptide, natively anchored to the viral membrane by the TM domain, recruits HR1 from the bilayer surface to form a C_3_-symmetric 6HB structure, suggesting a mechanism by which 6HB formation is coupled to membrane fusion.

## RESULTS

### HR1 binds to phospholipid bilayer

**Fig. 1.**
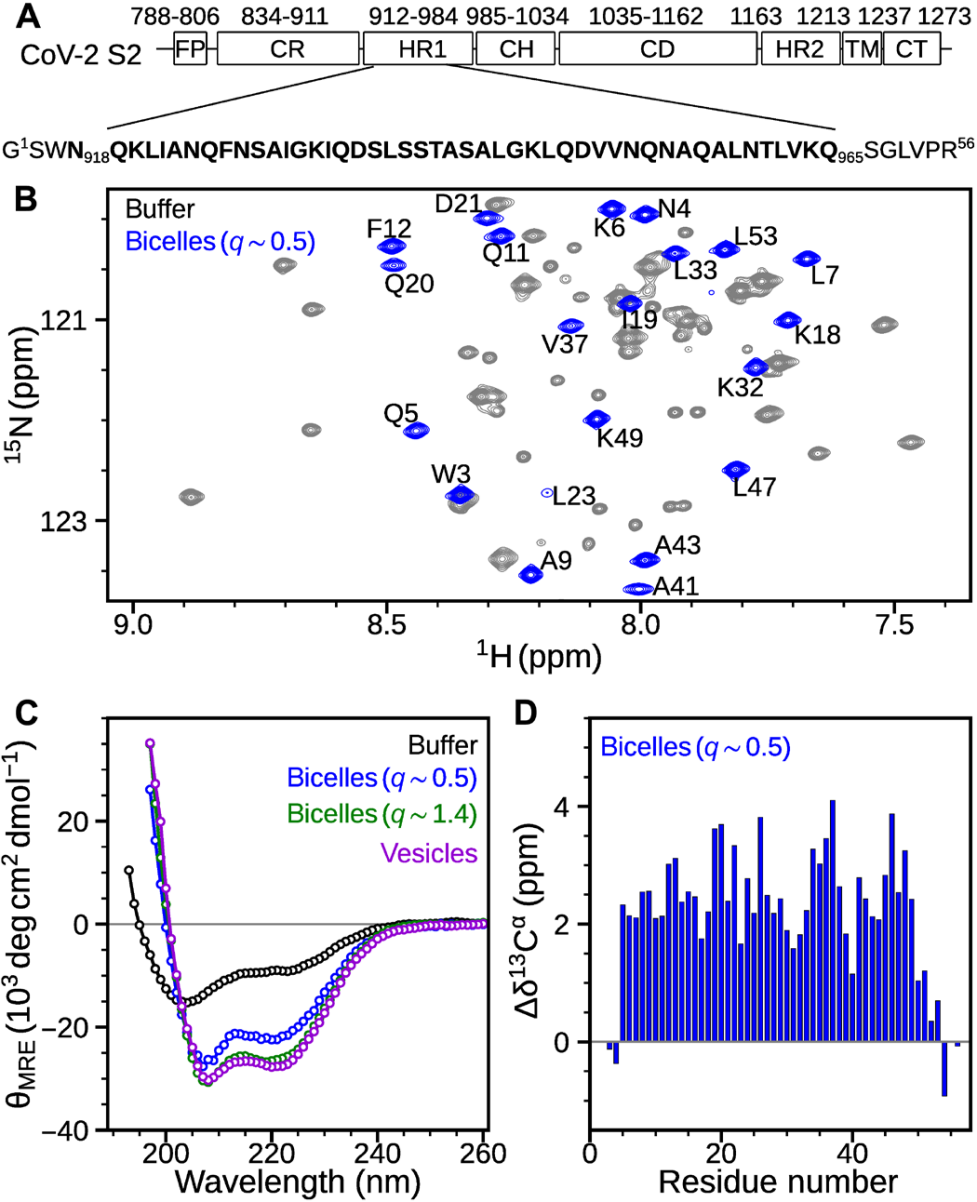
Phospholipid induced α-helicity in HR1. (**A**) Domain architecture of SARS-CoV-2 S2 protein with primary amino acid sequence of HR1 in bold. Residues 1 to 787 belonging to the S1 subunit of S protein are not shown. For brevity, HR1 residues are renumbered from 1 to 56. FP, fusion peptide; CR, connecting region; HR1, heptad repeat 1; CH, central helix; CD, connector domain; HR2, heptad repeat 2; TM, transmembrane; CT, cytoplasmic tail. (**B**) Overlay of small regions from the ^1^H-^15^N TROSY-HSQC NMR spectra of 350 μM HR1 in the absence (gray) and presence (blue) of 120 mM DMPC/DHPC (*q* ~ 0.5; i.e., 40 mM DMPC and 80 mM DHPC) bicelles at 900 MHz. Assignments are marked for the resonances in the presence of bicelles. (**C**) Far-UV CD spectra of 10 μM HR1 in buffer (black), 50 mM DMPC/DHPC (*q* ~ 0.5, blue), 50 mM DMPC/DHPC (*q* ~ 1.4, green), and 4 mM POPC/POPG/CHOL SUVs (purple). (**D**) Secondary Δδ^13^C^α^ chemical shifts of 350 μM HR1 in the presence of 120 mM DMPC/DHPC (*q* ~ 0.5). Data collected at 35°C in 20 mM sodium phosphate buffer (pH 6) containing 30 mM NaCl.

To obtain insights into membrane binding properties of the HR1 domain of SARS-CoV-2 spike protein, we recombinantly expressed and purified this domain (Fig. 1A). Earlier biophysical studies of the isolated HR1 domain from SARS-CoV and HIV-1 fusion proteins revealed its propensity to adopt a tetrameric state in solution, although the biological relevance of this association remains unclear (*19*–*21*). In the absence of HR2, the solvent-exposed hydrophobic HR1 residues at positions “e” and “g” of the helical wheel may energetically favor the formation of tetramer over trimer.

A two-dimensional (2D) ^1^H-^15^N TROSY-HSQC (Transverse Relaxation Optimized Spectroscopy–Heteronuclear Single Quantum Coherence) NMR spectrum of HR1 in solution reveals two sets of resonances, indicative of the presence of two conformations that are in slow exchange on the NMR chemical shift time scale (Fig. 1B and fig. S1A). While one set of resonances is very sharp and exhibits narrow ^1^H spectral dispersion [7.7 to 8.4 parts per million (ppm)], resonances of the second set are much broader and show wider spectral dispersion (7.4 to 9.0 ppm), indicating an equilibrium between an intrinsically disordered monomer and a structured oligomer. Size exclusion chromatography coupled with multiangle light scattering (SEC-MALS) analysis yields an average molecular mass of ca. 20 kDa (theoretical monomer mass = 5.9 kDa), which is somewhat higher than expected for a trimeric species (fig. S1C). Sedimentation velocity (SV) experiments also indicate the existence of a minor monomeric (~1.0 S) and a major tetrameric (~1.95 S) species (fig. S1D), consistent with earlier studies of the HR1 domain (*19*, *20*).

Addition of small bicelles [*q* ~ 0.5; i.e., mixed micelles consisting of a 1:2 molar ratio of DMPC (1,2-dimyristoyl-*sn*-glycero-3-phosphocholine) and DHPC (1,2-dihexanoyl-*sn*-glycero-3-phosphocholine)] or DPC micelles to HR1 results in large chemical shift changes in the NMR spectrum, indicating phospholipid induced structural changes in HR1 (Fig. 1B and fig. S1, A and B). Only a single set of resonances is observed in the presence of bicelles and micelles, pointing to a lipid-bound conformation. Moreover, HR1 chemical shifts are similar in bicelles and DPC micelles, suggesting similar structure in both membrane mimetics. Further, SV experiments validate the monomeric state of HR1 in the micelle-bound form (fig. S1E). To exclude the possibility that the detergent (DHPC) fraction of the DMPC/DHPC bicelles is responsible for this lipid binding, experiments were also carried out in the presence of SUVs (POPC:POPG:CHOL = 7:2:1). As expected, owing to the large increase in the rotational correlation time upon binding to SUVs, HR1 resonances are too broad to observe in TROSY-HSQC spectra at both pH 6 and pH 4, while several resonances belonging to the dynamically disordered His_6_-tag remain visible at pH 4 (fig. S2). This result concurs with our earlier observation using analogous peptides derived from HIV-1 gp41 (*16*): His_6_-HR1 resonances of gp41 broaden beyond the detection threshold upon addition of SUVs, indicative of similar membrane binding propensities of SARS-CoV-2 and HIV-1 for this segment of their ectodomains (fig. S3).

CD measurements were used to monitor secondary structural changes upon phospholipid binding. The far-ultraviolet (UV) CD spectrum for HR1 (10 μM) in solution depicted low α-helical ([θ]_222_ ≈ −12,000 deg cm^2^ dmol^−1^) content of ca. 29% (Fig. 1C). The α-helical content increased ([θ]_222_ ≈ −27,700 deg cm^2^ dmol^−1^) to ca. 84% in the presence of SUVs, indicating a membrane-bound α-helical structure of HR1 (henceforth termed mHR1). An increase in the helical content to ca. 67% ([θ]_222_ ≈ −22,000 deg cm^2^ dmol^−1^) upon addition of small isotropic tumbling bicelles (*q* ~ 0.5) supports a similar structural transition. To obtain residue-specific secondary structure information, ^13^C^α^ chemical shifts were measured in the presence of the small bicelles (*q* ~ 0.5). The ^13^C^α^ chemical shift deviations (Δδ^13^C^α^) from random coil values provide information about the local secondary structure (*22*). Positive Δδ^13^C^α^ values (2 to 4 ppm) for residues Q5-K49 in the presence of bicelles are indicative of α-helix (Fig. 1D), which is in good agreement with the helical content derived from CD spectroscopy. Overall, NMR, CD, and AUC measurements are all indicative of a monomeric membrane-bound state for HR1 in the presence of phospholipids.

### The mHR1 helix exhibits dynamic disorder

**Fig. 2.**
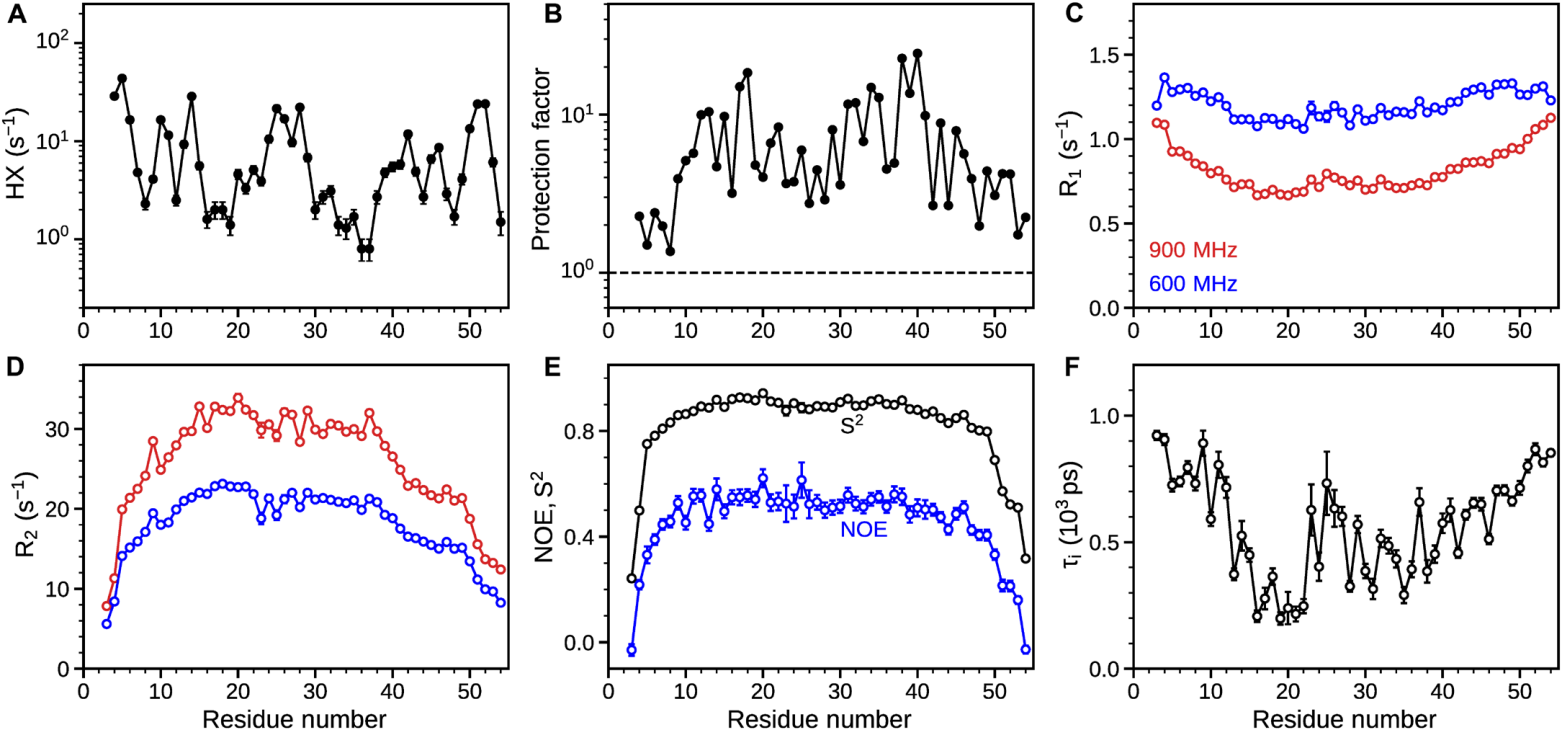
Dynamic helical conformation of mHR1. (**A**) Residue-specific HX rates of mHR1 at 30°C, normalized to pH 7. HX rates were obtained on 100 μM [^15^N/^2^H]-HR1 in the presence of 100 mM DMPC/DHPC. (**B**) Protection factors obtained as the ratio of intrinsic random coil exchange rates (*24*) and experimental HX rates at pH 7 (table S1). ^15^N backbone relaxation (**C**) R_1_, (**D**) R_2_ rates, and (**E**) ^15^N-{^1^H} NOE measured on 300 μM [^15^N/^2^H]-HR1 in the presence of 150 mM DMPC/DHPC at 600 MHz (blue) and 900 MHz (red). (E) Generalized order parameters (***S***^2^) for mHR1 are shown in black. (**F**) Time constants of internal motions of mHR1 derived from model-free analysis (*26*). See table S2 for backbone relaxation values.

Backbone amide protons exchange with solvent when they are not engaged in stable intra- or intermolecular hydrogen bonds (*23*). In a random coil polypeptide, i.e., in the absence of backbone amides forming H-bonds other than to water molecules, the intrinsic HX rate with solvent depends on pH and temperature in a well-characterized manner (*24*). The ratio of this intrinsic exchange rate and the rate observed in a protein is commonly referred to as the protection factor, *P*, where 1/*P* is the fraction of the time the intramolecular H-bond is absent. Near neutral pH, HX is base-catalyzed and increases 10-fold per unit of pH. The HX rates measured at pH 7 are 2.64-fold faster than at pH 6.55, validating the robustness of the measurement (fig. S4 and table S1). Rates normalized to pH 7 fall in the 0.5 to 50 s^−1^ range (Fig. 2A). Observed rates correspond to relatively low HX protection factors, *P*, in the range of 3 to 25 (Fig. 2B), although residues 8 to 49 would be expected to be engaged in α-helical H-bonds based on their ^13^C^α^ chemical shifts. An average *P* value of ca. 7 suggests that in mHR1, on average, each H-bond is broken about 14% of the time. However, quantitative interpretation of *P* values in terms of percentage H-bonding only applies to an aqueous environment. Measured *P* values are likely to be affected by the contacts with the lipid bilayer and, more conservatively, should be interpreted simply as an indicator of a substantial degree of dynamic disorder within the helical structure.

Further information on the backbone motions is obtained from ^15^N R_1_ and R_2_ (R_1ρ_) relaxation rates at 900 and 600 MHz ^1^H frequency, as well as ^15^N-{^1^H} heteronuclear nuclear Overhauser effect (NOE) values at 600 MHz. Residues in the central part of the mHR1 (residues N13-Q39) exhibit low R_1_ rates (ca. 0.7 s^−1^ at 900 MHz) and high R_2_ rates (ca. 30 s^−1^ at 900 MHz), while the N- and C-terminal regions (residues W3-F12 and N40-V54) show higher R_1_ and lower R_2_ rates, pointing to larger amplitude motions when approaching the termini (Fig. 2, C and D). Average R_2_/R_1_ ratios of about 17 and 35 at 600 and 900 MHz for the central part of mHR1 correspond to a rotational correlation time, τ_c_, of ca. 13 ns under the assumption of isotropic tumbling. In a well-packed globular protein, NOE values in the range of 0.75 to 0.85 are expected for a protein with τ_c_ ≈ 13 ns at 600 MHz. Hence, observed NOE values in the 0.5 to 0.6 range that fall well below this globular protein range also point to the presence of large internal motions throughout the mHR1 helix (Fig. 2E). Model-free analysis of the NMR relaxation data (*25*), optimized for an axially symmetric diffusion tensor (*26*), resulted in a global rotational correlation time (τ_c_) of 10.9 ns and anisotropy (*D*_||_/*D*_┴_) of 1.5. Generalized order parameters (*S*^2^) for residues Q5-K49 are in the range of 0.75 to 0.9, while the termini exhibit lower *S*^2^ values (0.4 to 0.7), pointing to increased flexibility. The fitted time constants for internal motions fall in the 200- to 1000-ps range, a time scale that is considerably longer than the typical values of 50 to 100 ps observed for small-amplitude motions in globular proteins (Fig. 2F).

### Solution structure of mHR1

**Fig. 3.**
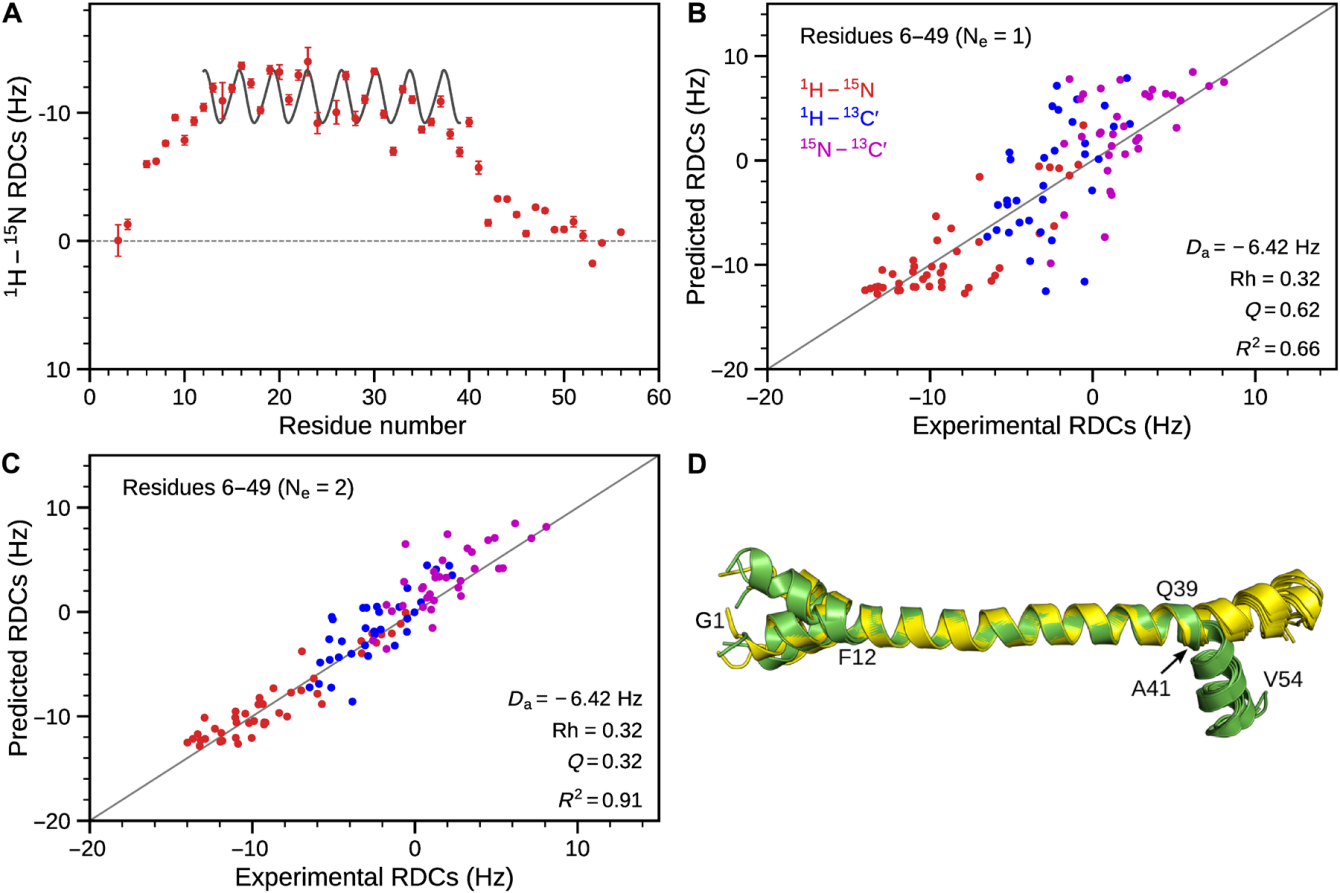
Solution structure of mHR1. (**A**) ^1^H-^15^N RDCs obtained in stretched positively charged polyacrylamide gel. Red circles represent experimental data, and the sinusoid corresponds to the best-fitted dipolar wave pattern (*28*) for residues N13 to Q39, with a root mean square deviation of 1.6 Hz against an idealized helical structure. Plotted values ignore the negative sign of γ(^15^N); i.e., |^1^J_NH_ + ^1^D_NH_| values are smaller than 90 Hz. Cross-validation SVD fits of experimental RDCs obtained in positively charged polyacrylamide gel against (**B**) a single best-fit structure and (**C**) a two-member ensemble structure. Structure calculations were carried out 44 times, each time excluding all RDCs for a given amide, and predicted RDCs for the amide were obtained from the lowest-energy structure. RDCs for ^1^D_NH_, ^1^D_NC′_ (one-bond ^15^N-^13^C′), and ^2^D_HC′_ (two-bond ^1^H-^13^C′) are shown in red, purple, and blue, respectively. ^1^D_NC′_ and ^2^D_HC′_ were upscaled by factors of 3.1 and 8.27, respectively, ignoring the effect of the sign of the ^15^N gyromagnetic ratio on the RDC, thereby ensuring that normalized RDCs of the same value correspond to the same orientational restraint. (**D**) Overlay of five lowest-energy RDC-refined structures of mHR1 displaying two distinct (yellow and green) conformations. A kink is observed at A41 for the ensemble fraction shown in green.

The ^13^C^α^ chemical shifts of mHR1, together with an uninterrupted string of strong sequential amide-amide NOEs, point to a contiguous α-helical structure (Fig. 1D and fig. S5), consistent with the high ellipticity observed in the CD spectrum (Fig. 1C). To detect any possible bends or kinks in this helix, orientational restraints were obtained by collecting RDCs under two slightly different alignment conditions, using stretched neutral and positively charged polyacrylamide gels (*27*). The sum of one-bond scalar and residual dipolar ^1^H-^15^N couplings, |^1^J_NH_ + ^1^D_NH_|, in the anisotropic media are smaller than 90 Hz, indicating that the N-H vectors are aligned roughly parallel to the static magnetic field (Fig. 3A). For residues N13-Q39, a clear sinusoidal oscillatory RDC pattern is observed with a periodicity of 3.6, but deviations from ideal helical behavior (*28*) exceed the experimental uncertainties (Fig. 3A). RDCs for this region span a narrow range from −8 to −14 Hz, indicating that the time-averaged helical axis orientation is tilted at an angle of ca. 15° relative to the magnetic field. Although the N-terminal region (residues K6-F12) appears to adopt helical structure based on ^13^C^α^ chemical shifts and NOEs, increased amplitude of motions toward the termini gradually attenuates the RDCs and results in disruption of the sinusoidal pattern. In contrast, a sharp drop in RDCs at N40-Q42 points to a possible kink in the helix. In addition, the C-terminal region (residues Q42-G52) displays smaller RDCs in the range of 0 to 3.5 Hz, despite strong α-helical propensity (Fig. 1C), indicative of increased dynamics in the helix. RDCs represent the time-averaged conformation over the entire picosecond-millisecond time scale, and internal motions that reorient internuclear N-H vectors result in attenuation of the corresponding RDCs (*29*).

To accurately determine the strength (*D*_a_) and rhombicity (Rh) of the alignment tensor, ^2^D_HC′_ (two-bond ^13^C′-^1^H^N^) and ^1^D_NC′_ (one-bond ^13^C′-^15^N) RDCs were also measured (fig. S6A and table S3). Agreement between experimental RDCs and a set of structural coordinates can be established by singular value decomposition (SVD) (*30*) and is commonly expressed as a quality factor, *Q* (*31*). With a *Q* factor of about 25%, RDCs (^1^D_NH_, ^2^D_HC′_, and ^1^D_NC′_) for residues N13-Q39 fit fairly well to an ideal α-helix (fig. S6C). Although 3_10_ H-bond patterns are rarely observed for polypeptides longer than about 10 residues, we also tested whether the experimental data fit to a 3_10_-helix (*Q* ≈ 46%), which proves not to be the case but demonstrates that RDCs can readily distinguish between α-helix and 3_10_-helix (fig. S6, C and F). While residues K6-K49 fit poorly (*Q* ≈ 58%) to a straight α-helix, separate fits for small regions at the N terminus (K6-F12) and C terminus (Q42-K49) display improved fits to α-helix with *Q* ≈ 44% and *Q* ≈ 25%, respectively (fig. S6). Reduction in the alignment strengths by about 1.4- and 2.3-fold for N-terminal and C-terminal regions, respectively, indicates that these regions sample multiple conformations relative to the central α-helix.

**Fig. 4.**
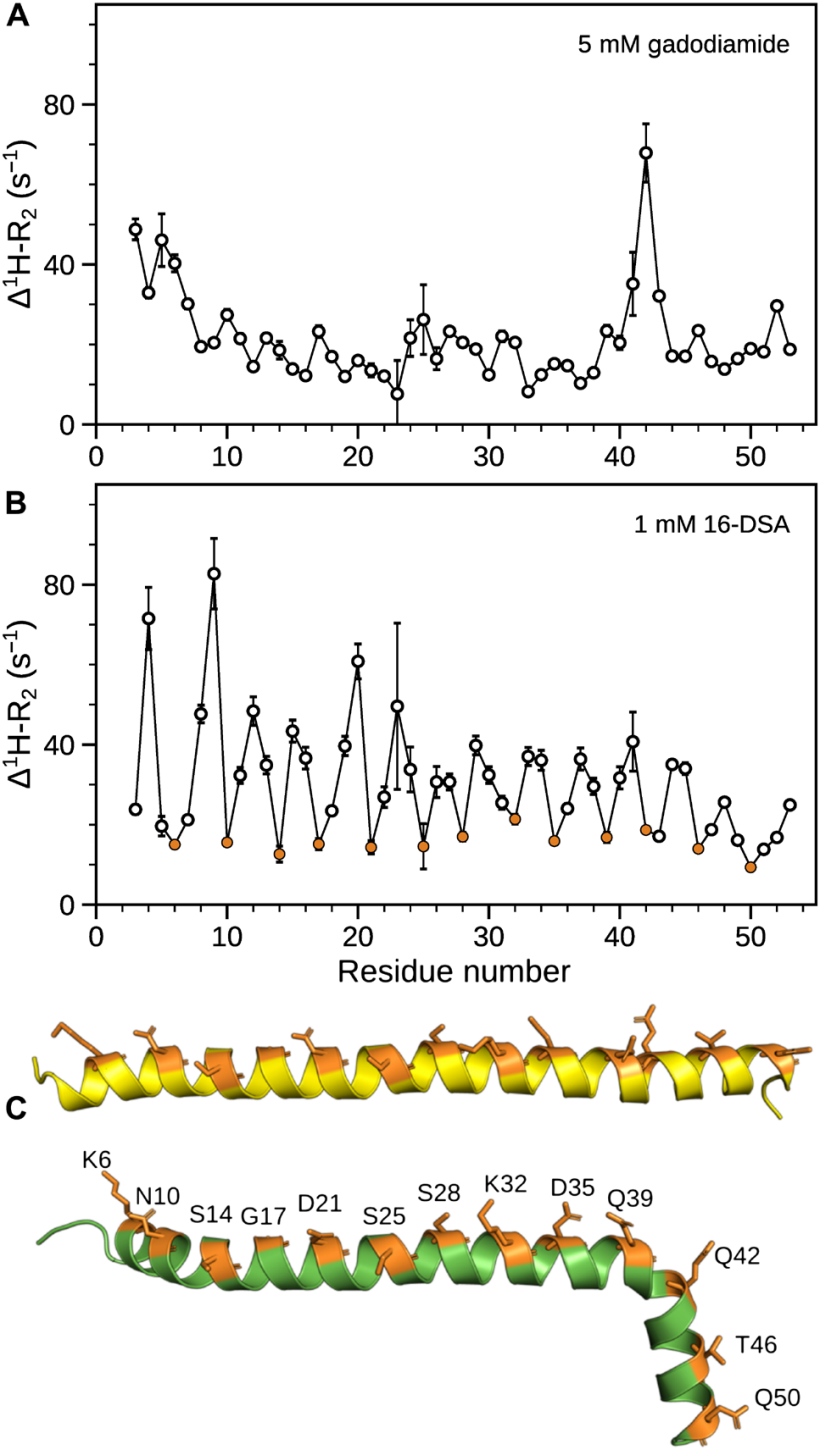
Membrane partitioning of mHR1. PRE rates (Δ^1^H-R_2_) of 80 μM [^15^N/^2^H]-HR1 in the presence of (**A**) 5 mM gadodiamide and (**B**) 1 mM 16-DSA. ^1^H-R_2_ rates were measured at 700 MHz in 20 mM sodium phosphate buffer (pH 6) containing 30 mM NaCl and 100 mM DMPC/DHPC at 30°C. Residues that experience an oscillatory pattern of minimal PRE rates are color-coded in orange. (**C**) Ribbon representations for the two conformations of mHR1, with side chains of residues with low PRE rates in the presence of 16-DSA shown as orange sticks. These polar hydrophilic residues correspond to the solvent-exposed face of mHR1.

Starting from a structure with ideal helical backbone torsion angles, structure calculations were performed using amide-amide NOEs and three types of RDCs from two alignment media as experimental restraints, supplemented with an H-bond potential of mean force (*32*). A conventional structure calculation, based on a single conformation approach, proved incompatible with the experimental data, as evidenced by poor RDC cross-validation (*Q* ≈ 60%; Fig. 3B). Note that when deriving *Q*, we simultaneously left out all RDCs measured for a given amide and repeated the structure calculation 44 times (K6-K49), each time omitting data for a different residue, thereby reporting a true cross-validation measure. To obtain a better structural model, ensemble calculations allowing two rapidly interconverting conformers were carried out (*33*, *34*). Residues N13-Q39 were restrained to a single structure, as this region fits well to a single, nearly straight α-helical structure with a sinusoidal RDC wave pattern, while the N- and C-terminal regions were allowed to move freely during the simulated annealing protocol. Calculations performed with an ensemble of two conformations resulted in an improved cross-validation with a *Q* ≈ 32% (Fig. 3C). While the N-terminal helical region displays somewhat flexible conformations, the C-terminal region primarily samples two distinct states. One set of structures adopts a nearly straight α-helix, and the second set displays a kink at A41 (Figs. 3D and 4C). Structure calculations performed by varying ensemble weights (from 20 to 40%) between the straight and kinked conformers resulted in slightly less convergence relative to equal, 50% ensemble weights.

### mHR1 partitions at the lipid-water interface

Paramagnetic relaxation enhancement (PRE) is a widely used tool to determine the membrane partitioning of peptides and proteins (*35*, *36*). Two paramagnetic probes, 16-doxyl stearic acid (16-DSA) and gadodiamide, which are confined to the interior of the lipid bilayer and the aqueous phase, respectively, were used to distinguish the membrane- and solvent-exposed residues of mHR1. For this purpose, PRE rates (Δ^1^H-R_2_) were obtained from the difference in ^1^H R_2_ rates measured under paramagnetic and diamagnetic conditions (Fig. 4 and fig. S7).

In the presence of the relatively bulky gadodiamide, whose closest approach to the backbone amides is restricted by the amino acid side chains, the observed PRE rates are fairly homogeneous and fall in the 10 to 25 s^−1^ range for most of the helix (Fig. 4A). The N-terminal region (W3-L7) and residues at the “kink” (A41, Q42, and A43) exhibit somewhat higher enhancement, indicating that water-dissolved gadodiamide can approach these backbone amides closer than those in the central part of the helix, consistent with the solution structure (Fig. 4, A and C). Kink residue Q42 experiences the highest PRE rate, caused by enhanced gadodiamide accessibility due to decreased protection from nearby backbone and side-chain atoms. In the presence of 1 mM 16-DSA, which on average corresponds to about 1 molecule of 16-DSA per bicelle, PRE rates in the range of 10 to 85 s^−1^ are observed (Fig. 4B). The modest relaxation enhancement observed even at the relatively high concentration of 16-DSA indicates that mHR1 does not deeply enter the interior of the bicelles but must be partitioned at the lipid-water interface. This conclusion is substantiated by a long stretch of mHR1 residues that exhibit an oscillatory Δ^1^H-R_2_ pattern of low PRE rates, with a periodicity of ~3.6 residues (Fig. 4B). The lipid-attached paramagnetic spin cannot approach these amides closely and mapping these residues on the mHR1 structure shows that this entire stretch is composed of hydrophilic amino acids (Fig. 4C). These data provide an exceptionally clear signature for a helix embedded at the lipid-water interface, where the solvent-exposed surface shows a much smaller PRE effect than the hydrophobic, lipid-facing residues.

While the PRE rates together with the solution structure provide residue-specific information on the interaction with the membrane, the nature of the kink at A41 may be affected by the use of the finite-sized bicelle, which at a *q* value of 0.5 is modeled to have a diameter of 42 Å for the planar bilayer component of the disk (*37*), which is too small to accommodate an α-helix longer than ca. 28 residues. Coincidentally, this 28-residue length matches the length of the N13-Q39 region, and this helical segment exhibits the regular sinusoidal RDC dipolar wave pattern (*28*), suggesting that the lipid affinity of the C-terminal segment could be responsible for the kink at residue A41. To evaluate whether the kink results from the limited size of the *q* = 0.5 bicelles, ^1^H-^15^N TROSY-HSQC spectra were acquired at *q* values ranging from 0.3 to 1.2, thereby varying the planar bilayer diameter from ~30 to 85 Å (fig. S8A). Although small chemical shift changes are present throughout the peptide sequence when increasing *q* (fig. S8B), by far, the largest chemical shift change is observed at the kink position for A41. An average increase in Δδ^13^C^α^ values of about 0.3 ppm in *q* = 0.5 over *q* = 0.3 bicelles indicates increased α-helicity for mHR1 with increasing bicelle diameter (fig. S8D), which is also evident from the CD spectrum. A modest increase (~12%) in α-helicity is observed when increasing *q* from 0.5 to 1.4 (Fig. 1C), suggesting that on a nearly flat cellular membrane mHR1 adopts a more regular α-helix. The presence of the kink at A41 when the bicelles are too small to accommodate the full-length helix points to decreased helical stability at this site, although the functional consequences of this lower stability remain unknown.

### HR2 adheres with low affinity to lipid bilayers

**Fig. 5.**
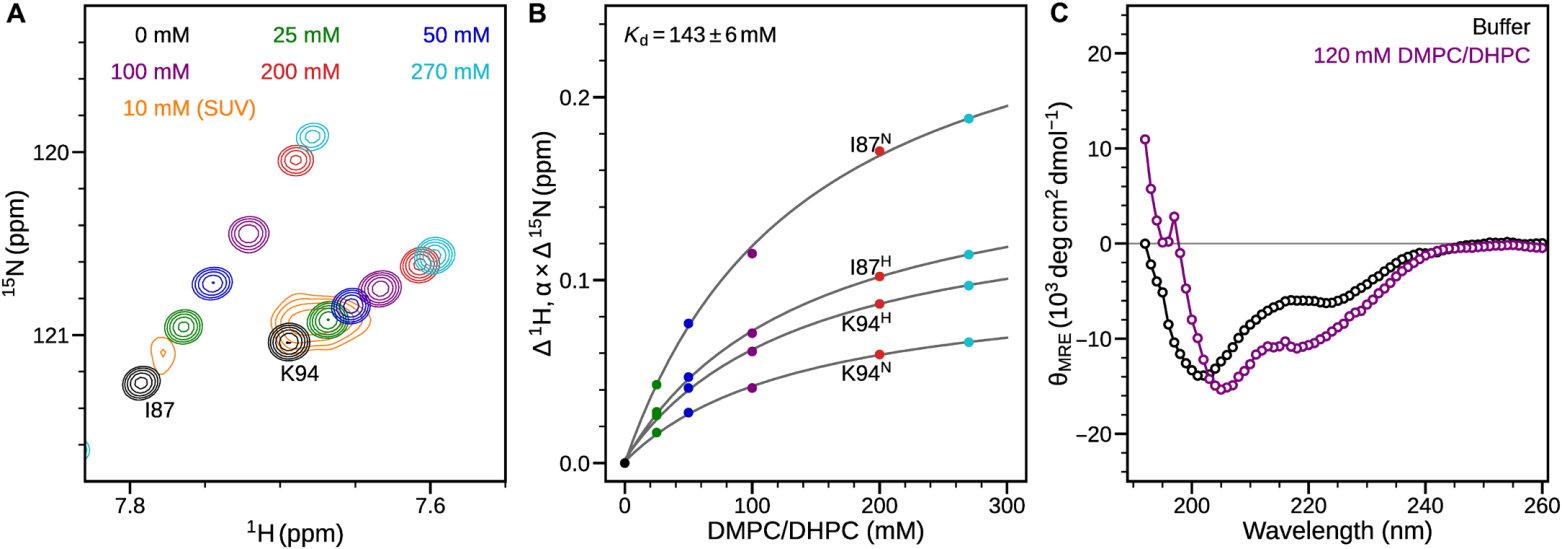
Membrane interaction of HR2. (**A**) Overlay of small regions from TROSY-HSQC spectra of 10 μM [^15^N/^2^H]-HR2 showing resonances for I87 and K94, in the presence of varying concentrations of DMPC/DHPC bicelles. The spectrum in the presence of 10 mM POPC/POPG/CHOL SUVs is shown in orange. (**B**) Plot of change in ^1^H and ^15^N chemical shifts across DMPC/DHPC concentrations. ^15^N chemical shift changes were scaled down by α = 0.14 for plotting purposes. Global fitting of the data for I87 and K94 chemical shifts resulted in a *K*_d_ of 143 mM. (**C**) Far-UV CD spectra of 10 μM HR2 in the absence (black) and presence (purple) of 120 mM DMPC/DHPC. Data obtained at 35°C in 20 mM sodium phosphate buffer (pH 6) and 30 mM NaCl.

Like HR1 in phospholipid-free buffer, a polypeptide corresponding to HR2 (residues 1171 to 1207 of S protein; Fig. 1A) also shows a concentration-dependent equilibrium between monomeric and oligomeric states (fig. S9, A and B). While there remains some ambiguity whether this state is trimeric or tetrameric (*38*, *39*), our SV data for the HR2 construct are in best agreement with a tetrameric arrangement (fig. S9C). The oligomerization affinity of HR2 is much lower than for HR1: At a concentration of 250 μM, the monomer population is about 10% for HR2 at 20°C, while HR1 is predominantly a tetramer at concentrations as low as 40 μM, under otherwise identical conditions (figs. S1D and S9C). At moderately elevated temperature (35°C) and low concentration (20 μM) of HR2, the equilibrium favors the monomeric state (fig. S9, A and B). A TROSY-HSQC spectrum of a 20 μM HR2 sample displays narrow linewidths for the major species, indicative of an intrinsically disordered monomer. Chemical shifts of HR2 change considerably with increasing amounts of bicelles (Fig. 5, A and B, and fig. S9D). The absence of substantial line broadening in the spectrum upon addition of bicelles, despite substantial chemical shift changes, indicates that the exchange rate between the free and lipid-bound state is fast (≥10^5^ s^−1^) on the NMR time scale, i.e., weak lipid binding. Some increase in linewidths is observed at the higher bicelle concentrations due to an increased population of the slower tumbling HR2-bicelle complex. A global fit of the ^1^H and ^15^N chemical shifts for well-isolated I87 and K94 resonances as a function of bicelle concentration yields a dissociation constant, *K*_d_ ≈ 143 mM (Fig. 5B). A far-UV CD spectrum shows low α-helical content of about 18% ([θ]_222_ ≈ −6000 deg cm^2^ dmol^−1^) for HR2 in the absence of lipids, consistent with intrinsically disordered structure (Fig. 5C). In the presence of 120 mM lipid bicelles, a large decrease in [θ]_222_ (−10,200 deg cm^2^ dmol^−1^) is observed, which is also accompanied by a shift in the minimum from 201 to 205 nm, indicative of an increase in the α-helical content to ca. 31%. Together with the *K*_d_ obtained from the NMR titration curve, these CD data suggest a total helical content of ca. 50% for HR2 in the lipid-bound state.

To ensure that the lipid binding observed for HR2 is not an artifact of the use of bicelles as a membrane mimetic, we also recorded NMR spectra in the presence of SUVs (Fig. 5A and fig. S9D), resulting in the strong attenuation of signals from residues that become immobilized when bound to the slowly tumbling vesicles. The resonances in the NMR spectrum shift in the same direction for both SUV and bicelle samples, indicating that HR2 adopts a similar structure when bound to SUVs and to bicelles. Although the much smaller change in chemical shifts upon addition of 0.7% (w/v) SUVs indicates that the equilibrium in the presence of SUVs remains strongly shifted to the disordered lipid-free state, the chemical shift change per mM lipid is comparable for SUVs and bicelles. While the lipid affinity of HR2 may appear low, we note that in the intact protein HR2 is anchored directly to the TM helix (Fig. 1A), which increases the effective lipid concentration it senses to ≥200 mM (Supplementary Text), suggesting that it will be predominantly lipid-bound when not otherwise restrained.

### Reconstitution of 6HB from mHR1

Although the above results demonstrate phospholipid-binding propensities for both HR1 and HR2, which we and others (*15*–*17*) propose to reflect a membrane-bound structural intermediate during the fusion process, it is challenging to trap this state in vivo due to its transient nature. Earlier biophysical and biochemical studies demonstrated the formation of 6HB by mixing equimolar concentrations of isolated HR1 and HR2 peptides in solution (*19*). We investigated whether the peptides also transition to the 6HB state in the presence of lipids, by titrating HR2 into the HR1-bicelle complex.

**Fig. 6.**
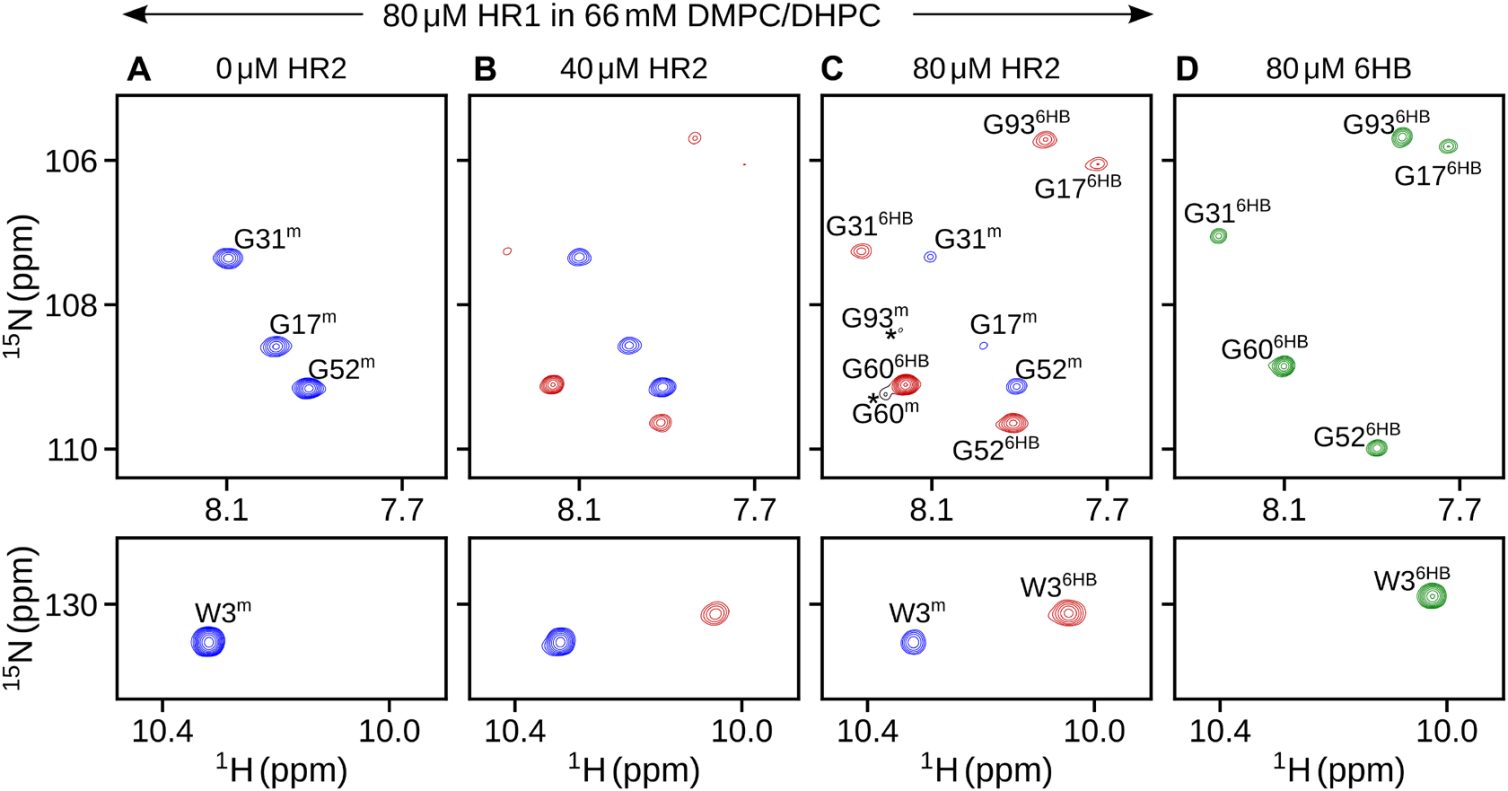
Reconstitution of 6HB from mHR1 and HR2. Small regions from ^1^H-^15^N TROSY-HSQC spectra of [^13^C/^15^N]-HR1 containing (**A**) 0 μM, (**B**) 40 μM, and (**C**) 80 μM [^13^C/^15^N]-HR2. Resonances corresponding to the membrane-bound (superscript “m”) and 6HB (superscript “6HB”) states are shown in blue and red, respectively. Minor population of HR2 (G60^m^ and G93^m^) bound to bicelles is marked by asterisks with contours in black (C). (**D**) Corresponding spectral region of [^15^N/^2^H]-6HB (green) in the absence of bicelles. Small chemical shift differences between 6HB resonances in (C) and (D) are attributed to ^2^H isotope effects and to change in chemical environment around the linker region. Data were collected at 800 MHz, 35°C, in 20 mM sodium phosphate buffer (pH 6) containing 30 mM NaCl and 66 mM DMPC/DHPC. Complete titration data are shown in fig. S10.

Titration was conducted by collecting a series of ^1^H-^15^N TROSY spectra of [^13^C/^15^N]-HR1 containing DMPC/DHPC in the presence of increasing concentrations of [^13^C/^15^N]-HR2. Figure 6A shows representative small regions for several well-resolved Gly (G17, G31, and G51, top) and Trp (W3 indole side chain, bottom) residues of mHR1 (colored blue). Addition of HR2 resulted in the appearance of a new set of resonances (colored red). Increasing the concentration of HR2 (up to 80 μM) resulted in the increase and decrease of intensities for red- and blue-colored resonances, respectively, indicative of switching of mHR1 to another state. Comparison of the final titration spectrum with that of 6HB (HR1-linker-HR2) shows that this new state corresponds to 6HB (Fig. 6D and fig. S10). Therefore, this titration demonstrates that addition of HR2 to the membrane-bound HR1 results in the formation of the postfusion 6HB state. At near-equimolar concentrations of HR1 and HR2, a third set of resonances (black) appear that correspond to the membrane-bound HR2 conformation (Fig. 6C and fig. S10), which highlights that the formation of 6HB represents a slow dynamic equilibrium between the lipid-bound states of HR1 and HR2 and the lipid-free 6HB.

### Comparison of solution and crystal 6HB structures

**Fig. 7.**
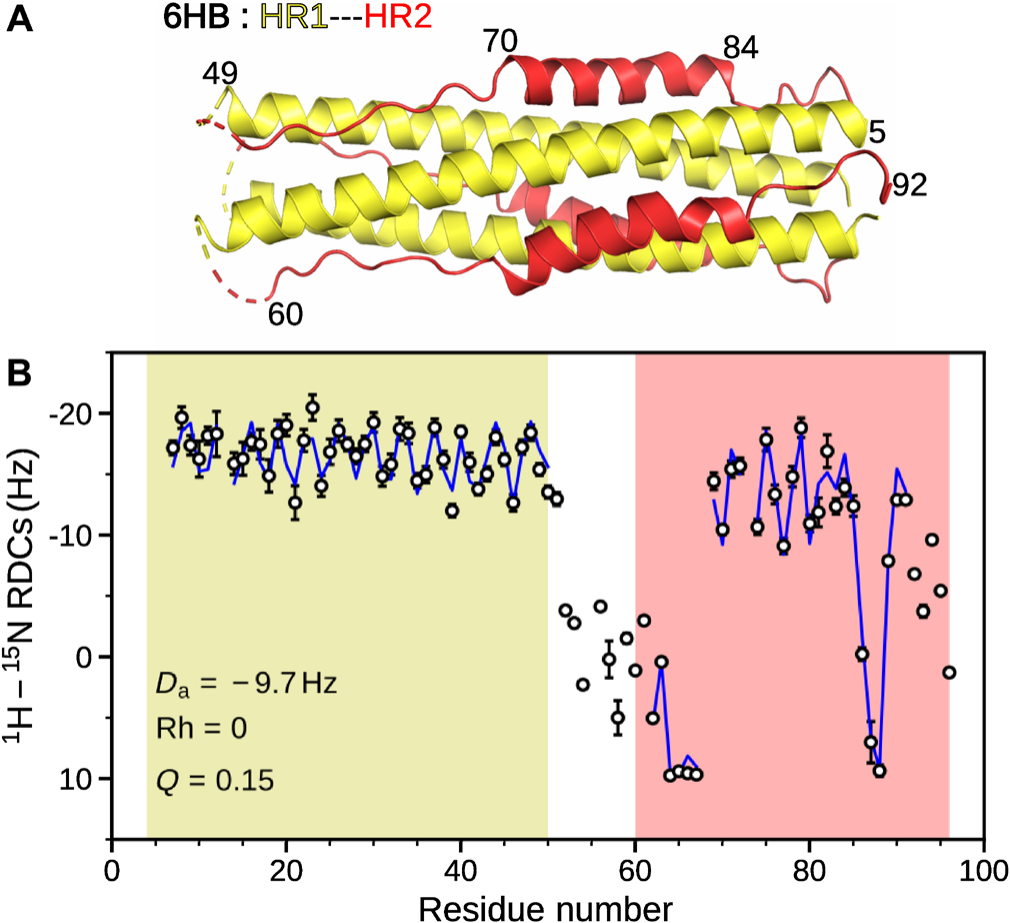
Characterization of the solution structure of 6HB. (**A**) Ribbon representation of the crystal structure of 6HB (PDB ID: 6M1V), with HR1 and HR2 shown in yellow and red, respectively. (**B**) Residue-specific ^1^H-^15^N RDCs were obtained in a stretched polyacrylamide gel [acrylamide, 4.17% (w/v) and bisacrylamide, 0.11% (w/v)] doped with positive charge [(3-acrylamidopropyl)-trimethylammonium chloride, 0.22% (w/v)]. Predicted RDCs from the x-ray structure are represented by blue lines. Experimental RDCs fit to the x-ray structure resulted in a *Q* factor of 15% (fig. S11D), indicating excellent agreement. Data collected at 800 MHz, 35°C, in 20 mM sodium phosphate buffer (pH 6), 30 mM NaCl.

The ^1^H-^15^N TROSY-HSQC spectrum of 6HB shows well-dispersed resonances, indicative of a folded structure (fig. S10). SEC-MALS and AUC experiments yielded molecular weights of 29.4 and 32.5 kDa, respectively, consistent with the 30.6-kDa mass expected for the homotrimeric 6HB (fig. S11, A and B). The nonnative designed linker region (residues L53-N62) exhibits Δδ^13^C^α^ values close to zero and appears dynamically highly disordered. Large ^13^C^α^ secondary chemical shifts (Δδ^13^C^α^) for residues Q5 to K49 of HR1 and Q69-N83 of HR2 regions coincide with α-helices in the 6HB x-ray structure (*40*), and negative Δδ^13^C^α^ values for residues A63-I68 point to backbone torsion angles in the β-region of Ramachandran space, with all Δδ^13^C^α^ values in good agreement with the x-ray crystal structure (Fig. 7A and fig. S11C). We note, however, that such Δδ^13^C^α^ values are qualitative markers for secondary structure and do not report on tertiary structure. By contrast, RDCs are exquisitely sensitive probes of both local and global protein structure. ^1^H-^15^N RDCs measured for the 6HB sample aligned in a stretched polyacrylamide gel exhibit the characteristic sinusoidal dipolar wave patterns (*28*) for residues L7-K49 and Q69-E84, as expected for α-helices (Fig. 7B and table S6). Furthermore, a low *Q* factor of 15% between the experimental ^1^H-^15^N RDCs and the 1.5-Å x-ray coordinates [Protein Data Bank (PDB) entry 6M1V] indicates that the structure present in solution is in excellent agreement with that observed in the crystalline state (Fig. 7B and fig. S11D).

## DISCUSSION

Fusion proteins of enveloped viruses catalyze fusion between the viral and host cell membranes, a prerequisite for host cell entry. Although these fusion proteins across different viruses (e.g., HIV-1, influenza, and SARS-CoV-2) vary widely in sequence, length, and target receptor recognition, they adopt a similar fusion mechanism (*3*). A successful fusion requires crossing of the substantial energy barrier posed by the repulsive hydration force, which steeply increases when the distance between the two membranes falls below 30 Å (*41*). In an analogous intracellular membrane fusion mechanism, SNARE (soluble *N*-ethylmaleimide–sensitive factor attachment protein receptor) proteins overcome this energy barrier through ATP (adenosine 5′-triphosphate) hydrolysis (*42*). Instead, viral fusion proteins undergo irreversible conformational changes from the metastable prefusion to the lowest-energy postfusion states, and the energy released during this process is used to transition the kinetic barrier of membrane fusion.

**Fig. 8.**
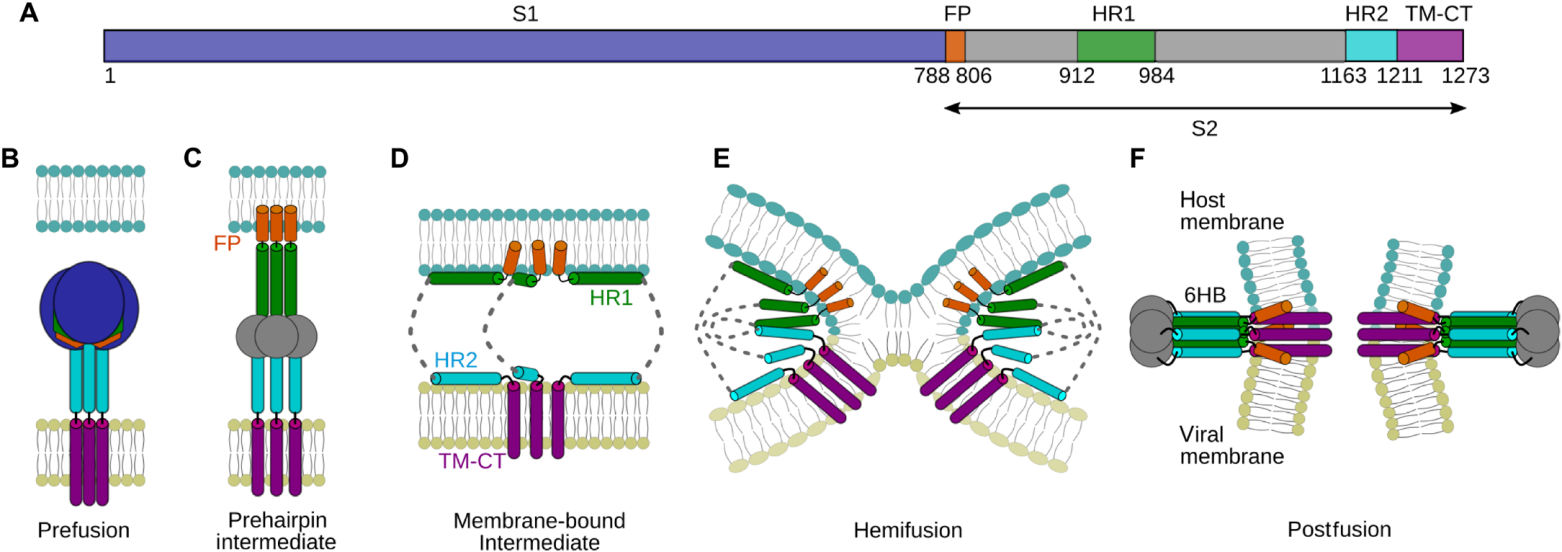
Proposed membrane fusion model. (**A**) Domain architecture of SARS-CoV-2 S protein with multiple domains and their boundaries. Domain coloring: FP, orange; HR1, green; HR2, cyan; TM-CT, purple; with the remainder of the S2 subunit shown in gray. Bottom panel (B to F) represents a cartoon model for membrane fusion mechanism. (**B**) Prefusion, (**C**) PHI, (**D**) membrane-bound intermediate, (**E**) hemifusion, and (**F**) postfusion states. The host and viral cell membrane are colored in teal and olive, respectively.

Several high-resolution structures obtained in pre- and postfusion states paved the way to understand the fusion process (*3*, *4*). However, lack of detailed structural information for the intermediate stages of the membrane fusion limits our understanding of this important biological phenomenon. In the extended PHI state, the HR1 and HR2 regions are presumed to adopt a linear stretch of trimeric helices, with FP and TM regions anchored in the host and viral membranes, respectively (Fig. 8C). While the membrane-bound intermediate state is not often represented in most fusion models, mounting evidence supports its existence (*13*–*17*).

In the present study, we used a construct that mimics the postfusion (6HB) state of the SARS-CoV-2 spike protein, formed by minimal lengths of HR1 and HR2 polypeptides that are connected by a small linker region to characterize the intermediate states of the membrane fusion process. Tertiary arrangement adopted by 6HB in solution (Fig. 7 and fig. S11) is in excellent agreement with the x-ray structure (*40*). However, when studied separately, these individual polypeptides, HR1 and HR2, exhibit a monomer-tetramer equilibrium in solution, consistent with earlier studies (*19*, *20*).

NMR and AUC studies show that HR1 adopts a monomeric membrane-bound conformation in the presence of phospholipid bicelles (Fig. 1 and fig. S1). Although HR2 has only weak affinity (*K*_d_ ≈ 143 mM) for lipids, it may interact with the viral membrane for a large fraction of time, due to its proximity to the TM domain (Fig. 8A). In the membrane-bound conformation, both HR1 and HR2 were found to adopt α-helical structures (Figs. 1 and 5C). The affinity of these regions for the phospholipid surface suffices to dissociate their intramolecular associations in the extended PHI state, leading to formation of lipid-bound states of HR1 and HR2 with host and viral membranes, respectively (Fig. 8D). Paramagnetic NMR studies demonstrate that these heptad repeats do not embed deeply in the bilayer but reside at the lipid-water interface (Fig. 4). The transition to these lipid-bound states pulls the viral and host cell membranes closer to one another while destabilizing the phospholipid bilayer surfaces (Fig. 8D). Attenuation of NMR resonances that accompany the transformation into α-helical structure in the presence of SUVs indicates that association of these HR regions with membrane bilayers is not influenced by the presence of detergents in the bicelles (Figs. 1 and 5A and fig. S2). Unlike HR2, which is proximal to the viral membrane, HR1 is distant from FP by about 100 amino acids (Fig. 8A). It is interesting to note that in addition to FP, a small region upstream to HR1, the internal FP (IFP) region (residues 891 to 906 of CoV-2), of SARS-CoV and CoV-2 was shown to embed in DPC micelles and other membrane mimetics (*43*–*45*). Insertion of IFP into the bilayer would bring HR1 in close proximity to the host cell membrane. Regions connecting FP-HR1 and HR1-HR2 (gray in Fig. 8) undergo substantial structural changes between their pre- and postfusion states (*4*), but their conformations in the intermediate states remain unknown. While the oligomeric state of the TM-CT region is debated for other class I fusion proteins (*46*–*50*), a recent study reported a C_3_-symmetric trimeric structure for the isolated TM domain of SARS-CoV-2 in isotropic bicelles (*51*). Intermolecular associations mediated by the hydrophobic residues in the TM domain may stabilize this homotrimeric arrangement of the fusion protein during the transient intermediate stages of fusion.

Our study of the structure of HR1 in the presence of small bicelles (*q* ~ 0.5) indicates that at least two conformations are required to fit the RDC data, with one conformation adopting a regular straight helix and the second conformation containing a kink at position A41 (Figs. 3 and 4). However, increased α-helicity in the presence of large bicelles (*q* ~ 1.4) and SUVs suggests that the α-helical structure becomes more regular on an extended flat bilayer (Fig. 1D). Membrane fusion requires strong local curvature of the lipid surfaces, and low helical stability at A41 may provide the flexibility needed for it to undergo conformational changes. Addition of HR2 to the membrane-bound state of HR1 resulted in the formation of 6HB, providing a plausible transition path from the membrane-bound intermediate state to the final postfusion state (Figs. 6 and 8F). However, before reaching this postfusion state, hemifusion represents another obligatory intermediate step (*52*), where the apposed, proximal leaflets of the bilayers are merged, while the distal leaflets remain intact (Fig. 8E). As our experiments are conducted on polypeptides that do not contain FP and TM regions and use isotropic bicelles, which can fuse edgewise and exhibit rapid lipid exchange during collisions in solution, the hemifusion state is not accessible in our study.

Our earlier work on HIV-1 gp41 in the presence of DPC micelles showed that its HR1 and HR2 regions are also lipophilic (*16*). Similar results on these gp41-HR regions in the presence of more native-like membrane mimetics (vesicles) support the relevance of these earlier studies (fig. S3). Overall, observation of membrane-bound states of the ectodomains of both HIV-1 and SARS-CoV-2 fusion proteins implies that this membrane-bound intermediate represents a conserved, obligatory step in the membrane fusion mechanism of class I fusion proteins.

## MATERIALS AND METHODS

### Recombinant protein expression and purification

6HB (fig. S10A) of SARS-CoV-2 fusion protein (GenBank ID: QHD43416.1) comprising HR1 (residues 918 to 965), a linker (SGLVPRGSG) bearing a thrombin recognition sequence, and HR2 (residues 1171 to 1207) were codon-optimized and cloned into pJ414 vector (ATUM). For the ease of purification, 6HB was flanked by an N-terminal polyhistidine-tag (His_6_) and separated by a TEV protease cleavage site from 6HB. The plasmid was transformed into *Escherichia coli* BL21 (DE3), grown at 37°C, and induced for expression at an optical density (600 nm) of 0.8 with a final concentration of 2 mM isopropyl β-d-1-thiogalactopyranoside for 4 hours. Cell pellet derived from 1 liter of culture was lysed by sonication in 100 ml of buffer A (50 mM tris-HCl at pH 8 and 6 M guanidine hydrochloride). The lysate was spun at 45,000*g* for 30 min at 18°C followed by subjecting the supernatant to Ni–nitriloacetic acid affinity chromatography (NAC) in buffer A. The bound fraction was dialyzed overnight against TEV buffer (25 mM tris-HCl at pH 8, 100 mM NaCl, 0.5 mM EDTA, 1 mM dithiothreitol, and 20 mM imidazole) including 5 mM *n*-dodecyl-β-d-maltopyranoside (from Anatrace), followed by the addition of TEV-His_6_ protease for 4 hours at room temperature. The digest was subjected again to NAC in buffer A to separate the cleaved product from the residual uncleaved protein and TEV-His_6_ protease. The flow-through was subjected to reversed-phase high-performance liquid chromatography (HPLC) on a POROS 20 R2 column (Thermo Fisher Scientific) and eluted using a linear gradient of 0 to 60% (v/v) acetonitrile solution containing 0.05% (v/v) trifluoroacetic acid. Aliquots of the peak fraction were stored at −70°C. Purity was verified by both SDS–polyacrylamide gel electrophoresis and electrospray ionization mass spectrometry.

HR1 (Fig. 1A) was obtained by incorporating a stop codon within the linker sequence of the 6HB gene construct and the expression/purification scheme as described above. HR2 was obtained by subjecting the His_6_-6HB to thrombin protease cleavage in 25 mM tris-HCl at pH 7.5, 100 mM sodium chloride, 2 mM calcium chloride, 20 mM imidazole, and 5 mM *n*-dodecyl-β-d-maltopyranoside, followed by NAC, and HPLC of the flow-through.

HPLC-purified 6HB, HR1, and HR2 were folded by dialysis in 50 mM sodium formate at pH 3, followed by 50 mM sodium acetate at pH 4, and finally against 20 mM sodium phosphate buffer at pH 6 containing 30 mM sodium chloride. Isotope labeling was achieved by growing the cells in M9/D_2_O medium supplemented with ^15^N NH_4_Cl and ^13^C-d7 d-glucose or ^12^C-d7 d-glucose as the nitrogen and carbon sources, respectively.

Phospholipid DMPC/DHPC bicelles were prepared by dissolving DMPC powder in DHPC solution. Total lipid ([DMPC] + [DHPC]) concentrations ranging from 25 to 270 mM were used in the study. Unless otherwise stated, the *q* value of bicelles, {[DMPC]/([DHPC] − [DHPC]_free_)}, is about 0.5, where [DHPC]_free_ is the monomer concentration (~7 mM) of DHPC that is in equilibrium between bicelle and buffer solution. Unless stated otherwise, all bicelle and lipid concentrations refer to the total concentrations of lipid molecules.

### Liposome preparation

Liposomes were prepared by weighing appropriate amounts of POPC, POPG, and cholesterol (ovine wool, >98%) in a glass vial and dissolved in chloroform/methanol (2:1) organic solution. All lipid reagents were purchased from Avanti Polar Lipids, unless stated otherwise. The solution was dried under a nitrogen stream to form a thin lipid layer on the glass surface and lyophilized overnight to remove trace amounts of chloroform and methanol. Lipid film was resuspended either in 20 mM sodium phosphate buffer (pH 6) containing 30 mM NaCl or in 20 mM sodium acetate buffer (pH 4), to result in a final concentration of 50 mM lipids (POPC/POPG/CHOL = 7:2:1, mol %). The lipid suspension was hydrated in buffer for about 1 hour, followed by five freeze-thaw cycles to obtain a homogeneous solution, which was further subjected to ultrasonication. The translucent lipid solution containing SUVs was spun at 10,000*g* for 20 min to sediment tiny bits of metal released by the sonicator tip. The particle size distribution of the SUVs was determined by dynamic light scattering (DLS) at 25°C with 1 mM lipid concentrations. DLS measurements performed on a Zetasizer Nano ZS instrument (at a wavelength of 633 nm) resulted in an average diameter of ca. 25 nm.

### Sedimentation velocity

SV experiments were conducted at 50,000 rpm and 20°C on a Beckman Coulter ProteomeLab XL-I analytical ultracentrifuge. Samples of 6HB, HR1, and HR2 prepared in 50 mM NaCl and 25 mM sodium phosphate buffer (pH 6.0) were loaded in 12-mm pathlength, two-channel centerpiece cells, and scans were collected using both the absorbance (280 nm) and Rayleigh interference (655 nm) optical detection systems. Samples of HR1 were also analyzed in the presence of 10 mM DPC (from Anatrace); here, a DPC-free matching buffer was used as a reference. Sedimentation data were time-corrected and analyzed in SEDFIT 16.1 (*53*) in terms of a continuous c(*s*) distribution of Lamm equation solutions with a resolution of 0.04 to 0.05 S and a maximum entropy regularization confidence level of 0.68. Solution densities ρ and viscosities η were calculated in SEDNTERP (*54*). The protein partial specific volume and absorbance extinction coefficient (ε_280_) were calculated in SEDNTERP based on the amino acid composition. When required, protein partial specific volume was corrected for ^2^H, ^15^N, and ^13^C isotopic composition. Absorbance and interference c(*s*) distributions were analyzed simultaneously using the membrane protein calculation module in GUSSI 1.4.1 (*55*) to obtain the protein and detergent contributions to the sedimenting complex. Sedimentation coefficients were corrected to standard conditions in water at 20°C, *s*_20*,w*_.

### Size exclusion chromatography–multiangle light scattering

Molar masses of 6HB and HR1 were analyzed by analytical SEC with inline MALS (Wyatt-925- H2HC, DAWN Heleos; Wyatt Technology Inc.), refractive index (Wyatt-215-TRXH; Wyatt Technology Inc.), and UV (Waters 2487; Waters Corporation) detectors. Samples were applied (125 μl) to a pre-equilibrated Superose-12 column (1.0 × 30 cm; GE Healthcare) and eluted at a flow rate of 0.5 ml/min at room temperature in 20 mM sodium phosphate at pH 6.0 and 30 mM NaCl. Molar masses were calculated using the Astra software provided with the instrument. Calculated masses of 6HB (65 μM injection) and HR1 (90 μM injection) are 29.4 and 19.6 kDa, respectively.

### Backbone chemical shift assignments

Backbone chemical shift assignments were carried out on a 0.4 mM [^13^C/^15^N/^2^H]-HR1 sample in a buffer containing 20 mM sodium phosphate (pH 6), 30 mM sodium chloride, 150 mM DMPC/DHPC, and 3% D_2_O. ^1^H^N^, ^15^N, ^13^C^α^, and ^13^C′ chemical shifts were obtained from TROSY-based HNCO and HNCA spectra recorded at 35°C on a 700-MHz Bruker Avance III spectrometer equipped with a triple-axis gradient TXI cryogenic probe. Further, a 3D ^1^H^N^-^15^N-^1^H^N^ NOESY-HMQC spectrum (τ_mix_ = 150 ms) was obtained at 800-MHz on a Bruker Avance II spectrometer equipped with a triple-axis gradient to cross-validate the assignments. Data were processed with NMRPipe (*56*) and analyzed using CCPNMR (*57*). The same approach was followed for obtaining ^1^H^N^, ^15^N, ^13^C^α^, and ^13^C′ chemical shifts of [^13^C/^15^N/^2^H]-6HB at 0.4 mM concentration at 35°C, in 20 mM sodium phosphate (pH 6), 30 mM sodium chloride, and 3% D_2_O.

### HX experiments

HX rates were obtained on 0.1 mM [^15^N/^2^H]-HR1 in a buffer containing 20 mM sodium phosphate (pH 6.55 and pH 7), 30 mM sodium chloride, 1 mM imidazole, 1 mM 2,2-dimethyl-2-silapentane-5-sulfonate, and 100 mM DMPC/DHPC. Rates were measured using the WEX-III TROSY experiment (*58*) with a recycle delay (d1) of 5 s over seven durations of the water inversion interval, ranging from 5 to 1000 ms. Measurements were carried out at 30°C on an 800-MHz ^1^H frequency spectrometer. Intrinsic random coil HX rates were obtained from the SPHERE web server (*24*). pH values of the samples for HX experiments were derived from imidazole ^1^H chemical shifts (*59*).

### Backbone relaxation experiments

^15^N spin-lattice (R_1_) and spin-spin (R_1ρ_) relaxation rates, and ^15^N-{^1^H} NOE data were collected at 35°C on a 0.3 mM ^15^N/^2^H-enriched HR1 sample in 20 mM sodium phosphate buffer (pH 6.0), 30 mM sodium chloride, and 150 mM DMPC/DHPC at 900- and 600-MHz, using TROSY-based heteronuclear experiments (*60*). The R_1ρ_ spectra were collected using a radiofrequency spin-lock field strength of 2 kHz, and R_2_ rates were extracted from R_1ρ_ after correcting for ^15^N offset effects (*61*). The ^15^N-{^1^H} NOE data were collected in an interleaved manner, where alternate free induction decays were collected with and without 8 s of proton saturation. Lipari-Szabo model-free analysis was performed using the Modelfree4 program (*26*), by optimizing the axially symmetric diffusion tensor.

### Residual dipolar couplings

Partial alignment of 0.15 mM [^13^C/^15^N/^2^H]-HR1 in 20 mM sodium phosphate buffer (pH 6), 30 mM sodium chloride, and 120 mM DMPC/DHPC was obtained in neutral acrylamide [4.87% (w/v)] and bisacrylamide [0.13% (w/v)] stretched gels. Positively charged gel contains (3-acrylamidopropyl)-trimethylammonium chloride [0.34% (w/v)], acrylamide [4.53% (w/v)], and bisacrylamide [0.13% (w/v)]. Gels were radially compressed from 4.9 to 4.2 mm diameter by means of a funnel, used for entry of the sample into the NMR tube. Amide (^1^H-^15^N) RDCs were measured using TROSY-based experiments from the difference of couplings under anisotropic and isotropic conditions (*62*). One-bond ^15^N-^13^C′ and two-bond ^1^H-^13^C′ RDCs were derived from the difference in splitting observed between E.COSY-TROSY spectra (*63*), obtained under anisotropic and isotropic medium.

### Structure calculation

Structures were calculated with the Xplor-NIH software package (*64*), using a simulated annealing protocol in which the temperature in the bath was cooled from 3000 to 25 K in steps of 5 K. Convergence of NMR-derived structures when starting from randomized initial models and using RDC restraints is a well-known problem. On the basis of the α-helical chemical shifts, we chose to start with backbone torsion angles set to φ = −62.5° and ψ = −42.5° for residues N4-Q50. Fitted experimental restraints included ^1^D_NH_, ^1^D_NC′_, and ^2^D_HC′_ RDCs from the positively charged and ^1^D_NH_ RDCs from neutral stretched polyacrylamide gel. Backbone hydrogen bonding geometries were restrained via a potential of mean force, HBPot (*32*). Initial estimates of alignment tensor magnitude (*D*_a_) and Rh were obtained by SVD fitting of the RDCs to an idealized α-helix. *D*_a_ and Rh were floated during the structure calculation to obtain optimum values. A final round of structure calculations was performed with optimized *D*_a_ and Rh values of −6.4 Hz and 0.32, and −7.2 Hz and 0.57, for positively charged and neutral polyacrylamide gel, respectively. Force constants for different types of RDCs in two different alignment media were 1 kcal Hz^−2^ mol^−1^ for ^1^D_NH_, 0.15 kcal Hz^−2^ mol^−1^ for ^1^D′_NC′_, and 0.25 kcal Hz^−2^ mol^−1^ for ^2^D′_HC′_, where ^1^D′_NC′_ = 8.27 × ^1^D_NC′_ and ^2^D′_HC′_ = 3.10 × ^2^D_HC′_ (i.e., ^1^D′_NC′_ and ^2^D′_HC′_ being the values normalized to the ^1^D_NH_ couplings) that yielded best cross-validation performance according to a grid searching procedure. The ^1^D_NH_ RDC force constant multipliers (and thereby the multipliers for the other types of RDCs) were ramped up with a constant multiplicative factor throughout the protocol from 0.1 to 5; i.e., at 25 K, the ^1^D_NH_ force constant was ramped up to 5 kcal Hz^−2^ mol^−1^. Amide-amide NOEs were ramped from 1 to 10 kcal mol^−1^ Å^−2^.

For ensemble (two conformations) structure calculations, backbone atoms of the residues N13-Q39 were restrained using the RAP potential (*65*) and equal weights were given to each ensemble member, while all other residues were allowed to move. A total of 100 structures were calculated and the 10 lowest-energy structures were selected for representation. Quality of the structures (*Q*) was evaluated by eliminating all RDCs corresponding to a given amide, one residue at a time, and repeating the structure calculation from the modified set of input RDC restraints and calculating

$$Q=\frac{\text{rms}(D_{\text{obs}}-D_{\text{pred}})}{{\left\{D_{a}^{2}[4+3{\text{Rh}}^{2}]/5\right\}}^{1/2}}$$

for the lowest energy structure, where *D*_obs_ and *D*_pred_ are the observed and predicted value of the omitted RDC, respectively, and rms is the root mean square function.

### CD spectroscopy

Far-UV CD spectra were acquired at 35°C in either 20 mM sodium phosphate buffer (pH 6) containing 30 mM NaCl or 20 mM sodium acetate buffer (pH 4) on a JASCO J-810 spectropolarimeter using a 0.1-cm pathlength cuvette. Measurements were performed with 10 μM peptide concentrations. For samples containing SUVs, lipid concentrations in the range of 3 to 5 mM were used. A total lipid concentration of 50 or 120 mM was used for samples containing DMPC/DHPC bicelles. The helical content was estimated on the basis of a [θ]_222_ value of −33,000 deg cm^2^ dmol^−1^ for a 100% helix (*66*).

### Paramagnetic relaxation enhancement

Gadodiamide (287 mg/ml) solution and 16-DSA (16-DOXYL-stearic acid) were purchased from GE Healthcare and Sigma-Aldrich, respectively. PRE measurements were performed on a sample of 80 μM [^15^N/^2^H]-HR1 containing either 5 mM gadodiamide or 1 mM 16-DSA, while a control diamagnetic sample did not contain any paramagnetic agent. Samples contained 20 mM sodium phosphate buffer (pH 6), 30 mM NaCl, and 100 mM DMPC/DHPC. Proton R_2_ rates were measured using seven relaxation delays (ranging from 0 to 32 ms) at 30°C on a 700-MHz ^1^H frequency spectrometer.

## Notes

View/request a protocol for this paper from *Bio-protocol*.
