# Supplementary material for: Transient lipid-bound states of spike protein heptad repeats provide insights into SARS-CoV-2 membrane fusion
Source: Sci Adv. 2021 Oct 8;7(41):eabk2226. doi: 10.1126/sciadv.abk2226 (PMC8500521; doi:10.1126/sciadv.abk2226)
Supplement: Supplementary file 1 — Supplementary Text Figs. S1 to S11 Tables S1 to S6 [file sciadv.abk2226_sm.pdf]

Science Advances
AAAS

Supplementary Materials for

Transient lipid-bound states of spike protein heptad repeats provide insights into SARS-CoV-2 membrane fusion

Sai Chaitanya Chiliveri*, John M. Louis, Rodolfo Ghirlando, Ad Bax*

*Corresponding author. Email: chaitanya.chiliveri@nih.gov (S.C.C.); bax@nih.gov (A.B.)

Published 8 October 2021, *Sci. Adv.* **7**, eabk2226 (2021)
DOI: 10.1126/sciadv.abk2226

This PDF file includes:

Supplementary Text
Figs. S1 to S11
Tables S1 to S6

Supplementary Text

Estimate of lipid concentration sensed by HR2

HR2 is directly anchored to the membrane by the transmembrane helix. The volume sampled by the 50-residue HR2 domain may be estimated from either the end-to-end distance of a random coil of 50 residues (*ca* 4.2 nm) or from the length of a 50-residue α-helix (*ca* 7 nm). Here we use the larger volume sampled by the assumption of HR2 adopting an α-helical structure, which corresponds to a hemisphere of radius r=7 nm, or a sampled volume of 718 nm^3. Using a standard lipid headgroup surface area of 0.5 nm^2, the hemisphere is exposed to 308 lipid headgroups, or an effective concentration of *ca* 700 mM. A lower bound of *ca* 280 mM for the effective lipid concentration is obtained for a model where HR2 is fully extended to a length of 17.5 nm. We note that the effective lipid concentration sensed by HR2 scales approximately inversely with the value chosen for r but does not account for the presence of non-lipid species embedded in the membrane which will lower the effective lipid concentration.

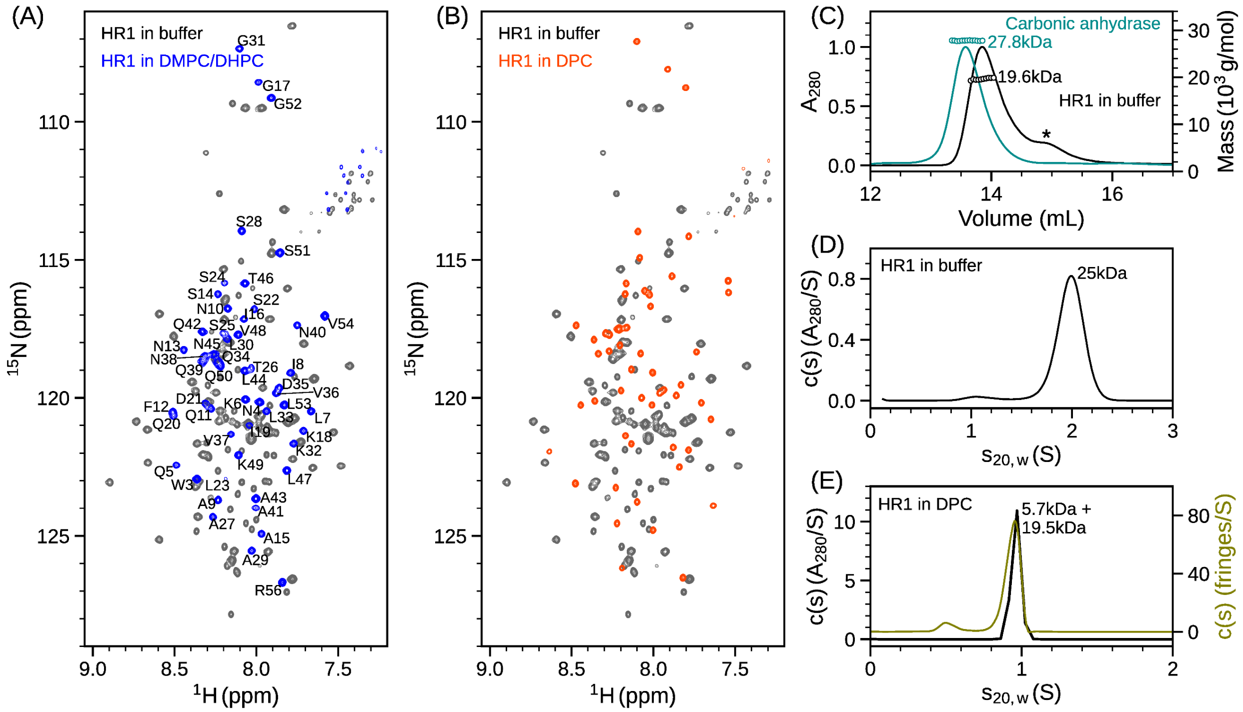

Fig. S1. Biophysical characterization of HR1. (A) ^{1}H-^{15}N TROSY-HSQC spectrum overlay of 50 µM [^{13}C/^{15}N]-HR1 in the absence (black) and presence (blue) of 100 mM DMPC/DHPC at 35 ºC. Residue specific assignments are shown for the resonances in the presence of bicelles. (B) ^{1}H-^{15}N TROSY-HSQC spectrum overlay of 50 µM [^{13}C/^{15}N]-HR1 in the absence (black) and presence (orange) of 100 mM DPC at 35 ºC. (C) Molar mass estimation by SEC-MALS indicates a major species of 19.6 kDa when injecting 125 µL of HR1 at 90 µM concentration on a 1 x 30 cm Superose-12 column at room temperature. The molar mass of the minor peak (*) could not be determined due to weak light scattering of the unstructured monomer (theoretical monomer mass = 5.9 kDa). Mass analysis of the protein standard (carbonic anhydrase, theoretical mass ~ 29 kDa) is shown in cyan color. (D) Sedimentation velocity absorbance c(s) profile for HR1 (40 µM) showing a major species at 1.95 S, 25 kDa tetramer. (E) Sedimentation velocity c(s) distribution for HR1 (120 µM) in the presence of 10 mM DPC using absorbance (black) and interference (olive) optics. Analysis resulted in a molar mass of *ca.* 25 kDa for HR1-DPC complex at 0.95 S, with DPC micelle and HR1 contributions of 19.5 kDa and 5.7 kDa, respectively, at 20 ºC.

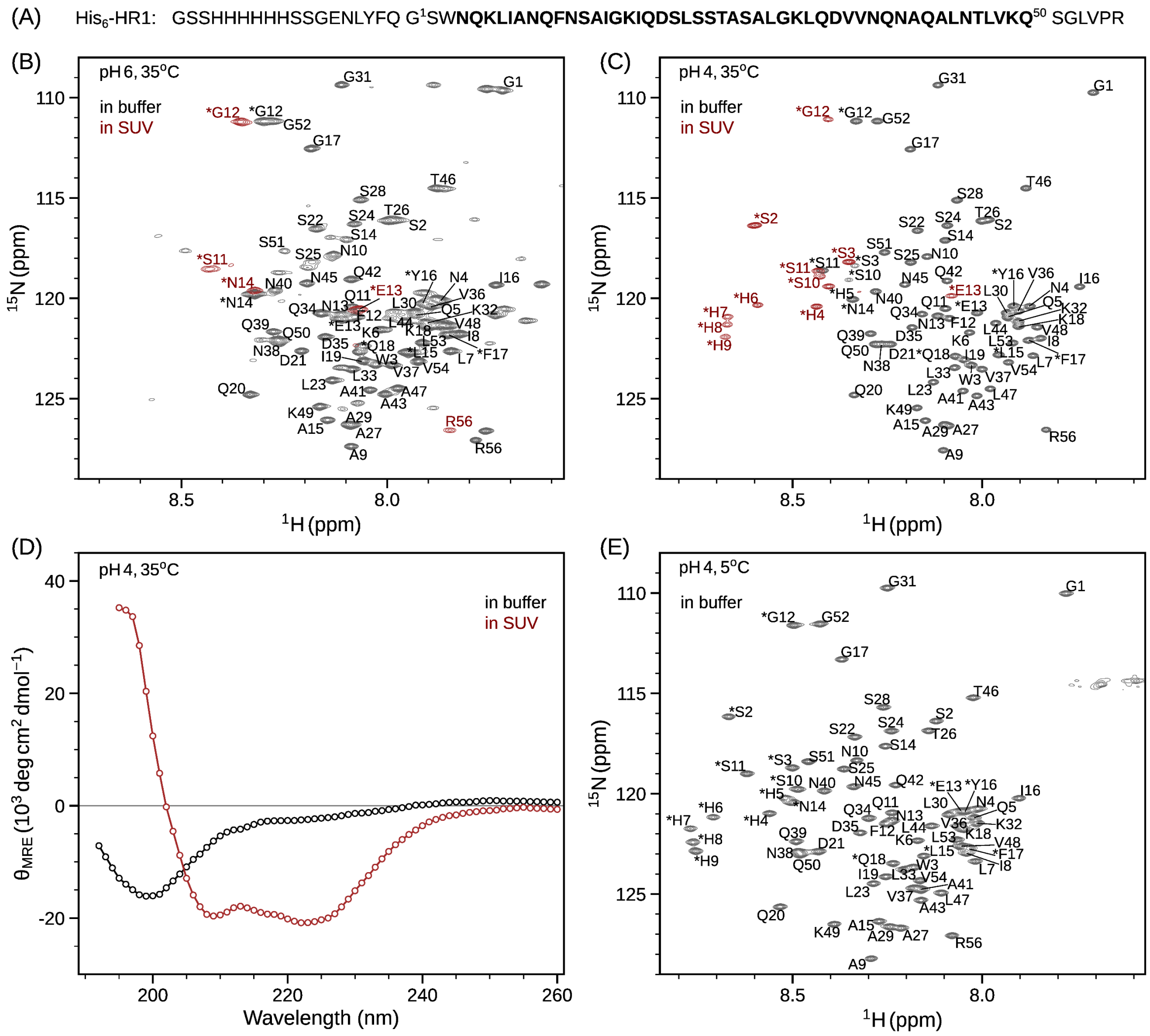

Fig. S2. His$_6$-HR1 interaction with phospholipid vesicles. (A) Primary amino acid sequence of His$_6$-HR1. Residues corresponding to HR1 are shown in bold. (B) ^{1}H-^{15}N TROSY-HSQC spectra overlay of 20 µM [^{15}N/^{2}H] His$_6$-HR1 in the absence (black) and presence (brown) of 10 mM SUVs in 20 mM sodium phosphate buffer (pH 6) containing 30 mM NaCl at 35 ºC. HR1 is in equilibrium between monomer and tetramer states. Assignments are marked for the monomeric state. Resonances from the histidine tag are marked with asterisks. (C) ^{1}H-^{15}N TROSY-HSQC spectra overlay of 20 µM [^{15}N/^{2}H] His$_6$-HR1 in the absence (black) and presence (brown) of 10 mM SUVs in 20 mM sodium acetate buffer (pH 4) at 35 ºC. HR1 predominantly adopts a monomeric conformation at pH 4. Assignments in the absence (black) and presence (brown) of SUVs are marked. His$_6$-tag residues in the absence of SUVs undergo exchange broadening at 35 ºC, and they appear at 5 ºC (panel E). Assignments for His$_6$-tag in the presence of SUVs were extrapolated from the spectrum obtained at 5 ºC (panel E). (D) Far UV CD spectrum of His$_6$-HR1 in the absence (black) and presence (brown) of 4 mM SUVs at pH 4 and 35 ºC. In the absence of SUVs, His$_6$-HR1 secondary structure resembles random coil, whereas it adopts α–helical structure (~64% helicity) in the presence of SUVs. (E) ^{1}H-^{15}N TROSY-HSQC spectrum of 20 µM [^{15}N/^{2}H] His$_6$-HR1 at 5 ºC in 20 mM sodium acetate buffer (pH 4). NMR spectra were collected at 600 MHz ^{1}H frequency spectrometer.

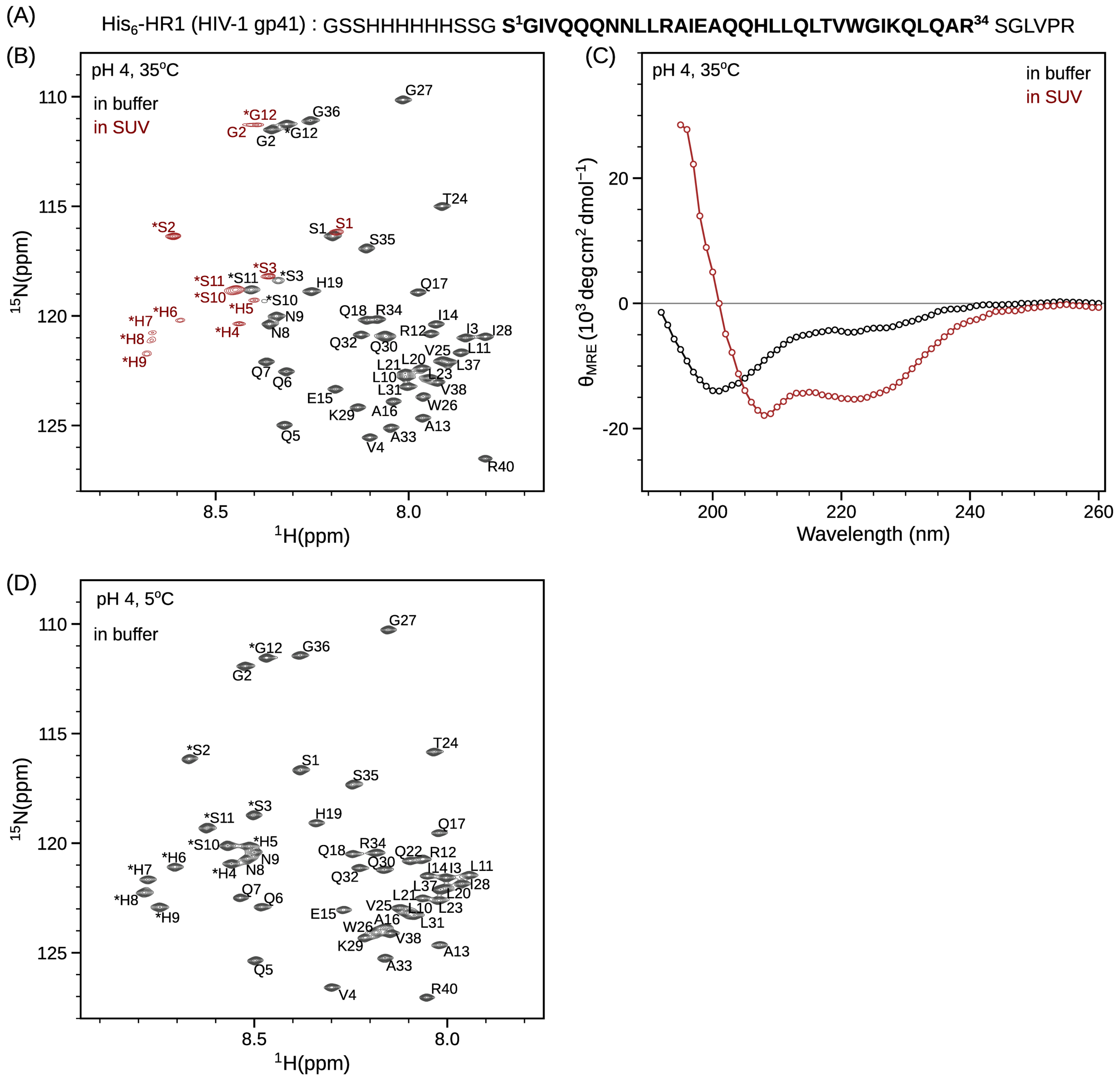

Fig. S3. HIV-1 gp41 His_6-HR1 interaction with phospholipid vesicles. (A) Primary amino acid sequence of HIV-1 gp41 His_6-HR1. Amino acids corresponding to HR1 are shown in bold. (B) ^1H-^{15}N TROSY-HSQC overlay of 20 μM [$^{15}N/^2H$] His_6-HR1 in the absence (black) and presence (brown) of 10 mM SUVs in 20 mM sodium acetate buffer (pH 4) at 35 ºC. Resonances from the histidine tag are marked with asterisks. (C) Far UV CD spectrum of His_6-HR1 in the absence (black) and presence (brown) of 4 mM SUVs at pH 4 and 35 ºC. His_6-HR1 in solution exhibits a small degree of α-helicity, whereas it adopts increased α-helical structure (~45% helicity) in the presence of SUVs. (D) ^1H-^{15}N TROSY-HSQC spectrum of 20 μM [$^{15}N/^2H$] His_6-HR1 at pH 4 and 5 ºC. His_6-tag residues undergo severe exchange broadening at 35 ºC but appear at 5 ºC. Assignments for His_6-tag resonances in the presence of SUVs were extrapolated from data obtained at 5 ºC (panel D). NMR spectra were collected at 600 MHz. As gp41-HR1 has low-solubility at pH 6, experiments were performed under acidic conditions.

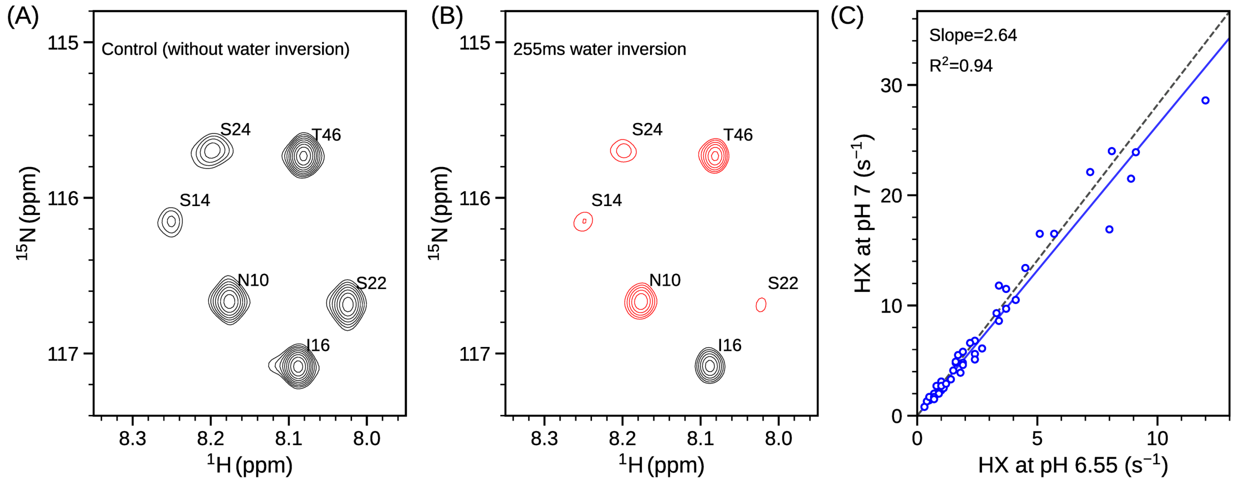

Fig. S4. Backbone amide HX rates of mHR1. (A) Excerpts from the ^{1}H-^{15}N TROSY-HSQC spectrum of mHR1, (A) without and (B) with a selective water inversion pulse. Black and red cross peaks represent positive and negative intensities, respectively. (C) Correlation plot of backbone HX rates obtained at pH 6.55 and pH 7. Rates obtained at pH 7 are 2.64-fold (solid blue line) faster than rates at pH 6.55, and a correlation coefficient (R^2) of 0.94 is obtained between these two datasets. A black dashed line represents a theoretical slope of 2.82. Data were obtained at 30 °C on 100 μM [^{15}N/^{2}H]-HR1 in 20 mM sodium phosphate (pH 6.55 or pH 7) buffer containing 100 mM DMPC/DHPC, 30 mM sodium chloride, 1 mM imidazole and 1 mM 2,2-dimethyl-2-silapentane-5-sulfonate.

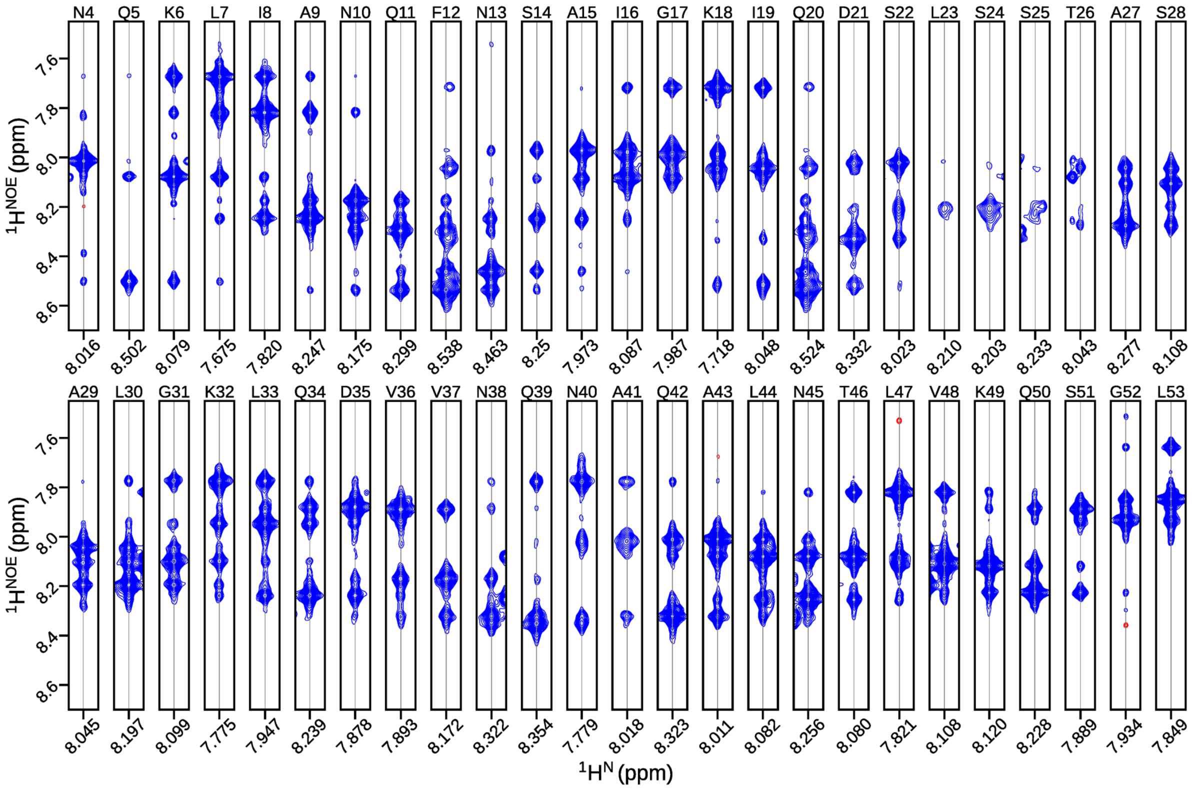

Fig. S5. NOE connectivity for mHR1. Strips extracted from a 3D NOESY-TROSY spectrum obtained on 300 µM [$^{15}N/^{2}H$]-HR1 in 150 mM DMPC/DHPC. Residues K6 to Q50 display sequential d_{NN} connectivities up to i±3 residues, characteristic of α-helical structure. Residues L23 to T26 display weaker diagonal peaks due to increased ^{1}H-R_2 rates (Fig. S7). Data collected on a 800 MHz spectrometer with 150 ms NOESY mixing time at 35 °C.

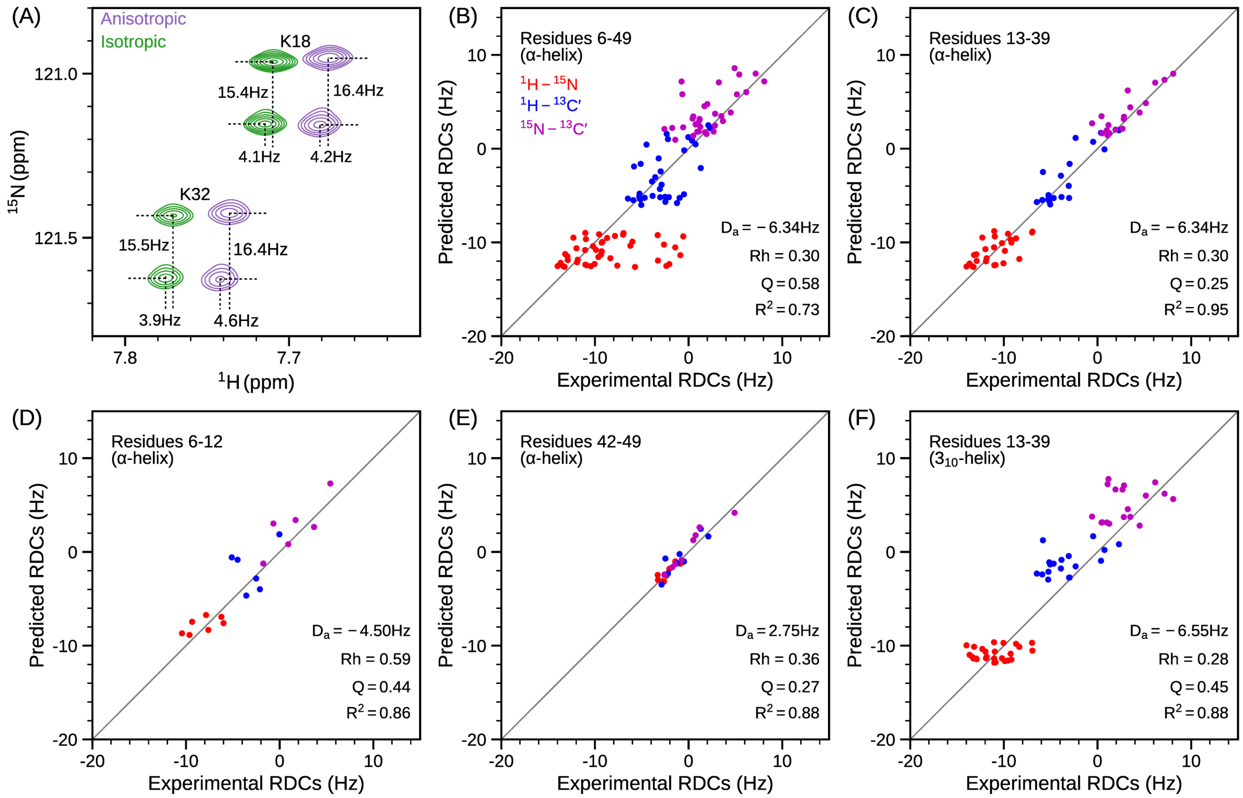

Fig. S6. RDCs to evaluate structural models of mHR1. (A) Overlay of small regions from the TROSY-HSQC-E.COSY spectra of isotropic (green) and anisotropic (purple) samples, used for extracting ^{1}H-^{13}C′ (two-bond) and ^{15}N-^{13}C′ (one-bond) RDCs. The anisotropic spectrum is moved by -0.03 ppm in the ^{1}H dimension for better visualization. SVD fits of experimental RDCs for residues (B) 6-49, (C) 13-39, (D) 6-12, and (E) 42-49 against ideal α-helix (backbone torsion angles, ϕ=-62.5° and φ=-42.5°). (F) SVD fit of experimental RDCs for residues 13-39 against a 3_{10}-helix (torsion angles, ϕ=-54° and φ=-27). ^{1}H-^{13}C′ and ^{15}N-^{13}C′ RDCs were derived from the difference in apparent J splittings measured under anisotropic and isotropic conditions. RDCs were normalized relative to ^{1}H-^{15}N values by upscaling them by factors of 3.10 and 8.27 for ^{1}H-^{13}C′ and ^{15}N-^{13}C′, respectively. Data for anisotropic RDCs were obtained using stretched, positively charged polyacrylamide gel at 800 MHz ^{1}H frequency, 35 °C.

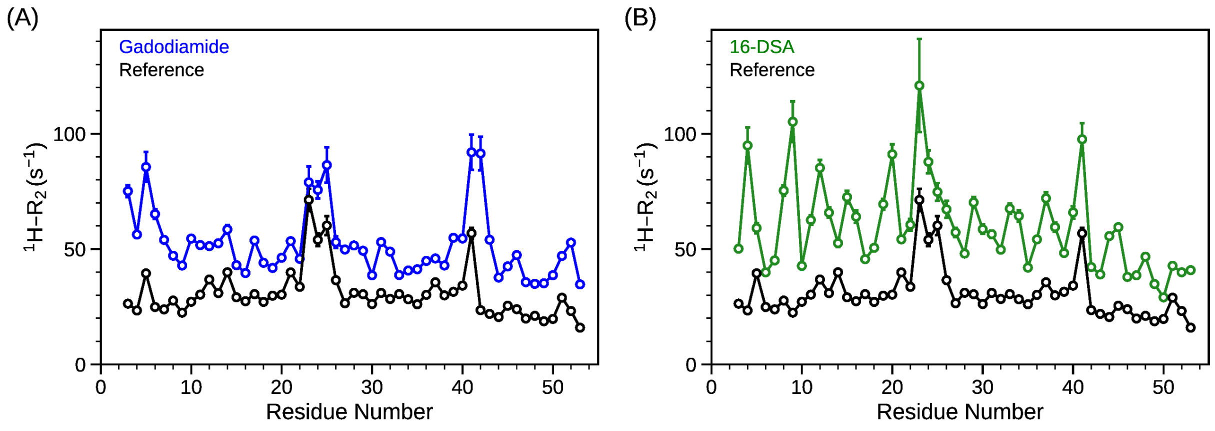

Fig. S7. mHR1 membrane partitioning. ^{1}H-R$_2$ rates in the presence of (A) 5 mM gadodiamide (blue) or (B) 1 mM 16-DSA (green). Rates for the diamagnetic reference are shown in black circles. Relaxation rates are obtained on a 700 MHz ^{1}H frequency spectrometer at 30 ºC on 80 µM [^{15}N/^{2}H]-HR1 in 20 mM sodium phosphate buffer (pH 6) containing 30 mM NaCl and 100 mM DMPC/DHPC.

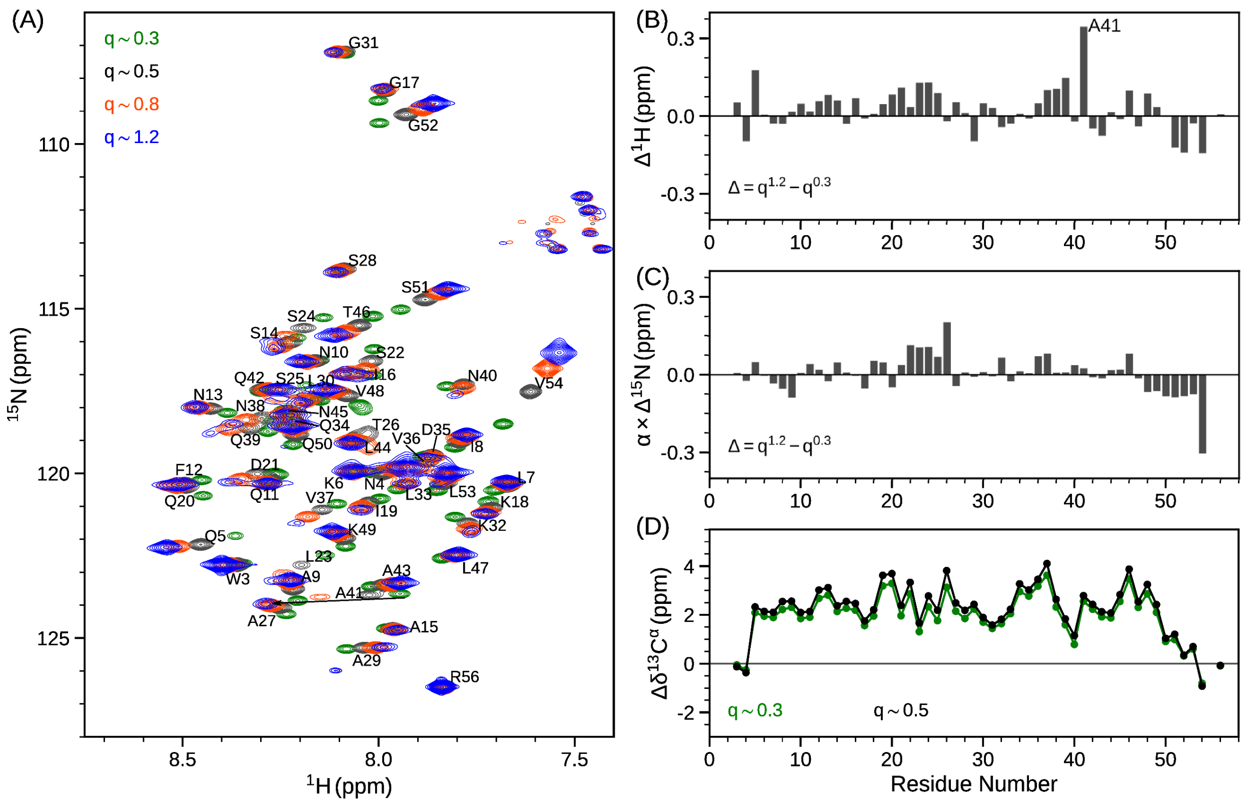

Fig. S8. Monitoring mHR1 chemical shifts by varying bicelle diameter. (A) ^{1}H-^{15}N TROSY overlay of mHR1 in DMPC/DHPC bicelles with different *q* values (0.3 – green, 0.5 – black, 0.8 – red and 1.2 – blue). Assignments are marked for the sample with *q* ~ 0.5. Increasing *q* beyond 1.2 resulted in further broadening of the resonances due to increased molecular weight of the complex. Change in (B) ^{1}H and (C) ^{15}N chemical shifts of HR1 (scaling factor, $\alpha = 0.14$), when varying the lipid ratio (and thereby bicelle size) from *q* ~ 1.2 to *q* ~ 0.3. (D) Deviation of $^{13}C^{\alpha}$ chemical shifts of mHR1 (*q* ~ 0.3 (green) and *q* ~ 0.5 (black)) from random coil chemical shifts. Data were collected in 20 mM sodium phosphate buffer (pH 6) and 30 mM NaCl at 35 °C.

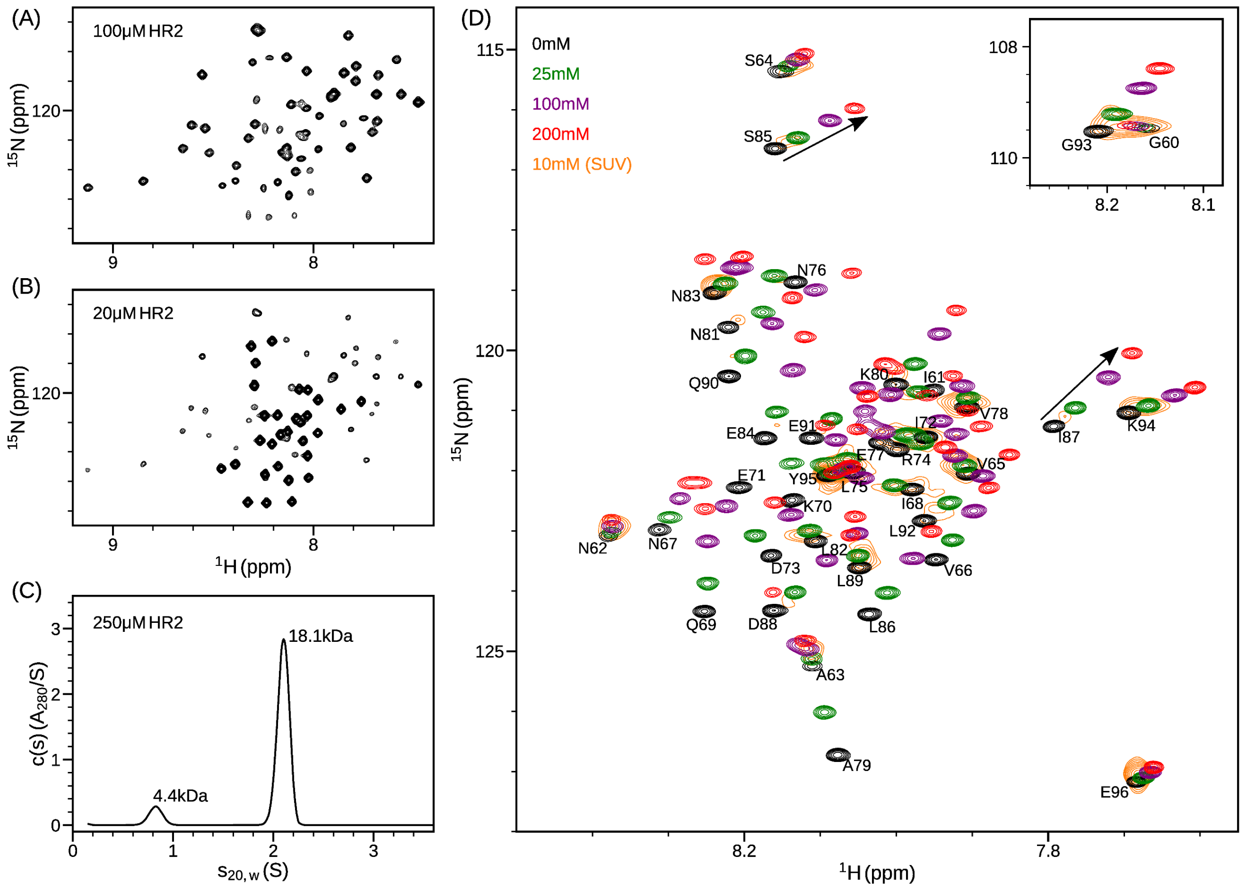

Fig. S9. Characterization of HR2. Excerpts from TROSY-HSQC spectra of (A) 100 μM and (B) 20 μM [^{13}C/^{15}N]-HR2 in 20 mM sodium phosphate (pH 6) and 30 mM NaCl at 35 °C. (C) Sedimentation velocity absorbance c(s) profile for HR2 (250 μM) showing the presence of a major 2.11 S, 18.1 kDa tetramer species and a minor 0.83 S, 4.4 kDa monomer species at 20 °C. Integration of distributions resulted in a population estimation of *ca* 10% for the monomer species. (D) Overlay of TROSY-HSQC spectra for 10 μM [^{15}N/^{2}H]-HR2 with varying concentrations (0 mM – black, 25 mM – green, 100 mM – purple, and 200 mM – red) of DMPC/DHPC bicelles. Glycine resonances are shown in the inset. A *q* of 0.5 is maintained for the titration experiments. A spectrum obtained in the presence of 10 mM POPC/POPG/CHOL (7:2:1) SUVs is shown in orange. Assignments are marked for the sample in the absence of bicelles. Data were collected in 20 mM sodium phosphate buffer (pH 6) and 30 mM NaCl. NMR spectra were obtained at 35 °C on a 600 MHz spectrometer. Monomer chemical shifts were assigned by obtaining ^{13}C$^{\alpha}$ chemical shifts obtained from a HNCA spectrum of a 50 μM [^{13}C/^{15}N/^{2}H]-HR2 sample containing 2 M Urea.

(A) 6HB - G[1]SW**NQKLIAN QFNSAIGKIQ DSLSSTASAL GKLQDVVNQN AQALNTLVKQ[50]** SGLVPR
GSG**G[60] INASVVNIQK EIDRLNEVAK NLNESLIDLQ ELGKYE[96]**

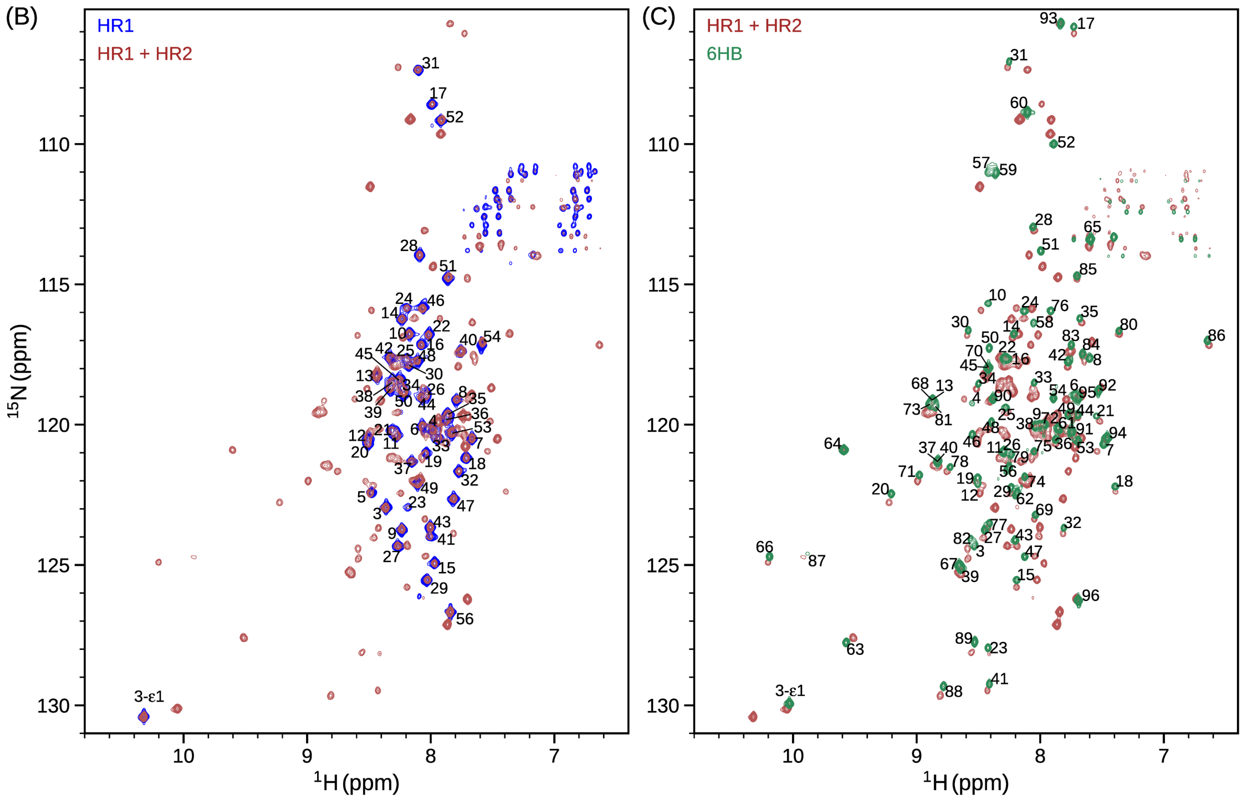

Fig. S10. Transition of mHR1 to 6HB. (A) Primary amino acid sequence of 6HB with native HR1 (residue 4-50) and HR2 (residue 60-96) regions shown in bold. (B) ^{1}H-^{15}N TROSY-HSQC spectrum of 80 μM [^{13}C/^{15}N]-HR1 (blue) in 66 mM DMPC/DHPC bicelles. The spectrum for a sample containing a final concentration of 60 μM [^{13}C/^{15}N]-HR2 added to the HR1-bicelle complex is shown in brown color. (C) Overlay of ^{1}H-^{15}N TROSY-HSQC spectra of 80 μM [^{15}N/^{2}H] 6HB in the absence (green) of bicelles over HR1+HR2 in the presence (brown) of bicelles. Small chemical shift differences between 6HB and HR1+HR2 samples containing bicelles are attributed to ^{2}H isotope effects and to change in chemical environment around the linker region.

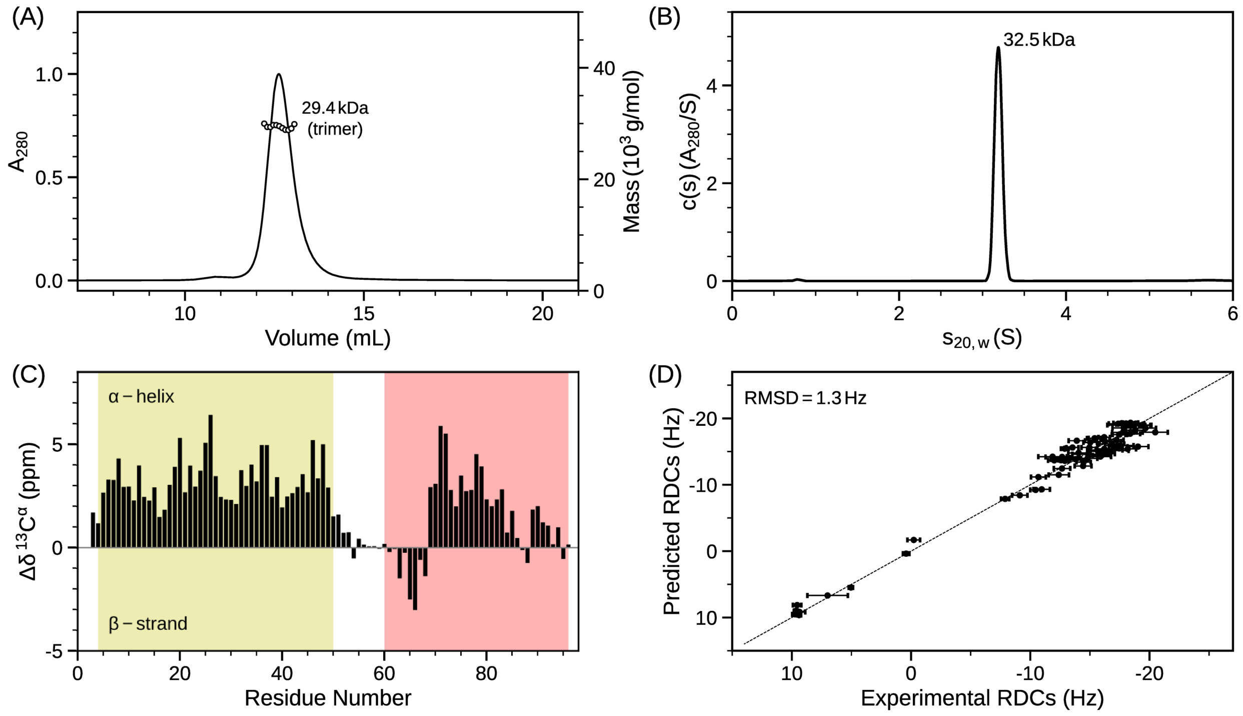

Fig. S11. Characterization of 6HB in the solution state. (A) Molar mass estimation by SEC-MALS indicated a single species corresponding to a trimer of 29.4 kDa. 6HB (125 µL injection at 230 µM) was fractionated on a 1 x 30 cm Superose-12 column. (B) Sedimentation velocity absorbance c(s) profile for 6HB (65 µM) showing the presence of a 3.17 S, 32.5 kDa trimer. (C) Plot for deviation of $^{13}C^{\alpha}$ chemical shifts from neighbor-corrected random coil shifts with HR1 and HR2 regions color coded in transparent yellow and red, respectively. Positive and negative $\Delta\delta^{13}C^{\alpha}$ values indicate α-helical and β-strand propensity. (D) A high degree of correlation is observed between experimental and predicted RDCs with a root-mean-square deviation of 1.3 Hz. NMR data were obtained on an 800 MHz ^{1}H frequency spectrometer at 35 °C in 20 mM sodium phosphate buffer (pH 6), 30 mM NaCl.

Table S1. Hydrogen exchange (HX) rates measured on 100 µM HR1 in the presence of 120 mM DMPC/DHPC in 20 mM sodium phosphate buffer (pH 6.55 and 7.0) and 30 mM NaCl at 30 °C obtained on an 800 MHz spectrometer.

Residue	HX at pH 6.55 (s^{-1})	HX at pH 7.0 (s^{-1})
1	-	-
2	-	-
3	17.9	-
4	10.7	28.8
5	16.5	-
6	5.7	16.5
7	1.9	4.8
8	1.0	2.3
9	1.5	4.1
10	5.1	16.5
11	3.7	11.5
12	1.1	2.5
13	3.3	9.3
14	1.02	28.6
15	2.4	5.6
16	0.7	1.6
17	0.7	2.0
18	0.7	2.0
19	0.5	1.4
20	1.9	4.6
21	1.4	3.3
22	2.4	5.1
23	1.8	3.9
24	4.1	10.5
25	8.9	21.5
26	8.0	16.9
27	3.7	9.7
28	7.2	22.1
29	2.4	6.8
30	0.9	2.0
31	1.0	2.7
32	1.0	3.1
33	0.5	1.4
34	0.4	1.3
35	0.5	1.7
36	0.3	0.8
37	0.3	0.8

38	0.8	2.7
39	1.6	4.8
40	1.7	5.5
41	1.9	5.8
42	3.4	11.8
43	1.6	4.9
44	1.0	2.7
45	2.2	6.6
46	3.4	8.6
47	1.2	2.9
48	0.7	1.7
49	1.5	4.1
50	4.5	13.4
51	9.1	23.9
52	8.1	24
53	2.7	6.1
54	0.7	1.5

Table S2. ^{15}N backbone relaxation data of HR1 in 100 mM DMPC/DHPC, 20 mM sodium phosphate (pH 6) and 30 mM NaCl, 35 °C, measured at 600 and 900 MHz ^{1}H frequency. Generalized order parameter (S^2) and internal motion time scales (τ_i) are obtained from Lipari-Szabo model-free analysis.

Residue	$^aR_1$ (Hz)	$^aR_2$ (Hz)	$^bR_1$ (Hz)	$^bR_2$ (Hz)	^{b}NOE	S^2	τ_i (ps)
3	1.10	7.84	1.20	5.60	-0.03	0.24	922
4	1.08	11.32	1.36	8.41	0.22	0.50	905
5	0.93	19.93	1.28	14.11	0.33	0.75	725
6	0.93	21.38	1.29	15.16	0.39	0.78	740
7	0.90	22.51	1.30	15.93	0.45	0.81	795
8	0.85	24.13	1.26	17.12	0.46	0.83	731
9	0.84	28.46	1.28	19.45	0.53	0.86	891
10	0.80	24.89	1.22	18.00	0.45	0.86	591
11	0.81	26.45	1.25	18.30	0.55	0.88	805
12	0.76	27.94	1.20	19.92	0.56	0.89	716
13	0.71	29.63	1.12	20.99	0.45	0.89	373
14	0.73	29.71	1.12	21.43	0.58	0.92	525
15	0.73	32.80	1.12	22.03	0.50	0.89	449
16	0.67	30.12	1.08	21.87	0.55	0.92	207
17	0.68	32.79	1.12	22.84	0.55	0.93	277
18	0.70	32.37	1.12	23.13	0.56	0.92	364
19	0.67	32.23	1.09	22.81	0.54	0.92	199
20	0.67	33.89	1.12	22.71	0.62	0.94	239
21	0.68	32.41	1.09	22.79	0.53	0.91	216
22	0.69	31.72	1.06	21.84	0.53	0.91	247
23	0.76	29.84	1.19	18.81	0.53	0.88	627
24	0.72	30.56	1.13	21.31	0.51	0.91	403
25	0.80	29.17	1.13	19.21	0.61	0.89	733
26	0.77	32.11	1.20	21.20	0.52	0.88	633
27	0.75	31.78	1.16	22.01	0.53	0.89	602
28	0.73	28.38	1.08	20.22	0.50	0.89	325
29	0.75	32.28	1.18	22.02	0.51	0.89	570
30	0.70	29.91	1.11	21.16	0.52	0.91	386
31	0.71	29.36	1.12	21.35	0.56	0.92	315
32	0.76	30.63	1.18	21.12	0.53	0.90	514
33	0.72	30.42	1.14	20.88	0.51	0.90	486
34	0.71	29.64	1.16	20.72	0.54	0.91	434
35	0.71	29.98	1.16	21.07	0.55	0.92	291

36	0.72	29.11	1.15	19.87	0.51	0.90	393
37	0.74	31.99	1.22	21.27	0.56	0.90	658
38	0.73	29.70	1.16	20.84	0.55	0.92	385
39	0.78	27.88	1.19	19.25	0.48	0.88	453
40	0.78	26.54	1.17	18.83	0.51	0.88	575
41	0.82	24.89	1.22	17.52	0.50	0.86	627
42	0.82	22.89	1.22	16.53	0.50	0.87	457
43	0.86	23.22	1.27	16.33	0.47	0.85	608
44	0.86	22.34	1.29	15.90	0.43	0.83	655
45	0.87	21.70	1.31	15.48	0.49	0.85	650
46	0.86	21.31	1.26	15.02	0.51	0.86	512
47	0.91	22.42	1.32	15.85	0.42	0.81	704
48	0.91	21.03	1.32	15.02	0.41	0.80	705
49	0.95	21.34	1.33	15.13	0.41	0.80	662
50	0.94	18.75	1.26	13.44	0.33	0.69	714
51	1.00	15.56	1.26	11.16	0.21	0.57	800
52	1.06	13.69	1.30	9.94	0.21	0.52	867
53	1.08	13.24	1.31	9.66	0.16	0.51	815
54	1.13	12.41	1.23	8.26	-0.03	0.32	853

Table S3. Backbone RDCs of HR1 in the presence of DMPC/DHPC. Experimental uncertainties in the measured couplings are 0.7 Hz ($^1D_{NH}$), 0.2 Hz ($^1D_{NC'}$), 0.3 Hz ($^2D_{HC'}$). Couplings (in Hz) are listed according to the residue number of the amide ^{1}H or ^{15}N and ignore the negative sign of the ^{15}N gyromagnetic ratio.

Residue	$^1D_{NH}{}^a$	$^1D_{NH}{}^b$	$^2D_{HC'}{}^b$	$^1D_{NC'}{}^b$
6	-5.6	-6.0	-1.2	-0.2
7	-8.0	-6.2	0.0	0.7
8	-8.8	-7.6	-0.8	0.1
9	-8.7	-9.6	-0.7	0.4
10	-8.8	-7.9	-1.5	-0.1
11	-11.4	-9.4	-1.7	0.2
12	-10.7	-10.4		
13	-10.7	-12.0	-1.7	0.1
14	-13.2	-11.0		
15	-14.0	-11.9	-1.0	0.3
16	-13.7	-13.7	-1.0	0.5
17	-12.6	-12.3	-1.9	0.1
18	-13.6	-10.2	0.1	1.0
19	-13.5	-13.4	-1.7	0.2
20	-12.0	-13.2		
21	-12.4	-11.0	-0.8	0.4
22	-14.1	-12.9	-1.0	0.4
23	-14.0	-14.0		
24	-10.9	-9.2		
25				
26	-12.3	-10.0		
27	-12.1	-12.9	-1.9	0.1
28	-7.6	-9.6	-0.2	0.2
29	-11.8	-11.1	-0.2	0.7
30	-13.4	-13.2	-2.1	0.1
31	-7.5	-9.9	-1.5	0.2
32	-7.3	-7.0	0.7	0.9
33	-13.2	-11.8	-1.3	0.3
34	-9.6	-11.0	-1.7	0.3
35	-6.6	-8.7		
36	-8.7	-9.3		
37	-10.6	-10.9	-1.2	0.1
38	-7.2	-8.4	-1.6	-0.1
39	-6.1	-7.0	0.2	0.6
40	-13.0	-9.3	-1.0	0.2
41	-5.4	-5.7		
42	-1.2	-1.4	-0.3	-0.2
43	-3.3	-3.3	0.7	0.6
44	-4.7	-3.3	-0.9	-0.3

45	-3.0	-2.1	-0.8	0.1
46	-0.5	-0.6	0.4	0.1
47	-3.5	-2.6	-0.7	-0.1
48	-4.0	-2.4	-0.2	0.1
49	-1.1	-0.9	-0.4	-0.2

a – Neutral stretched polyacrylamide gel.

b – Positively charged stretched polyacrylamide gel.

Table S4. Structural statistics for 10 lowest energy structures of mHR1[a].

RMSD for distance, orientation and dihedral angle restraints (no. of restraints)	
NOE (97)	0.01Å
Dihedral angles[b] (96)	3.1°
RDCs[b] (154)	
Positively charged stretched gel	
N-H (43)	0.5 Hz
N-C′ (34)	1.2 Hz
H-C′ (34)	0.9 Hz
Neutral stretched gel	
N-H (43)	0.3 Hz
RDC cross validation, Q_{free}[b]	32%
Backbone RMSD (over residues 13-49)	
Regular α-helix conformation	0.4 Å
Kinked α-helix conformation	0.4 Å
Ramachandran Statistics[b]	
Most Favored	100%
Additionally allowed	0%

[a] Structures were deposited in the PDB with accession code: 7R95
[b] Residues 6-49

Table S5. Proton R_2 rates of 80 μM [$^{15}N/^{2}H$]-HR1 in the presence of 100 mM DMPC/DHPC obtained at 700 MHz ^{1}H frequency spectrometer at 30 °C. R_2 rates under paramagnetic conditions are obtained either in the presence of 5 mM gadodiamide or 1 mM 16-DSA. Buffer conditions are 20 mM sodium phosphate (pH 6) and 30 mM sodium chloride.

Residue	$R_{2,\ \mathrm{Diamagnetic}}$ (s^{-1})	$R_{2,\ \mathrm{Gadodiamide}}$ (s^{-1})	$R_{2,\ \mathrm{DSA}}$ (s^{-1})
3	26.3	75.1	50.2
4	23.4	56.3	94.9
5	39.5	85.5	59.1
6	24.9	65.1	39.9
7	23.9	54.0	45.1
8	27.7	47.1	75.4
9	22.4	42.9	105.2
10	27.2	54.6	42.7
11	30.2	51.7	62.6
12	36.8	51.3	85.2
13	30.9	52.5	65.8
14	39.9	58.5	52.6
15	29.1	43.0	72.5
16	27.4	39.6	64.0
17	30.5	53.7	45.6
18	27.1	44.1	50.6
19	29.8	41.8	69.4
20	30.2	46.2	91.1
21	39.9	53.4	54.2
22	33.6	45.7	60.5
23	71.3	78.9	120.9
24	54.0	75.6	87.8
25	60.2	86.4	74.7
26	36.5	52.9	67.2
27	26.5	49.8	57.2
28	31.0	51.5	48.0
29	30.4	49.3	70.2
30	26.2	38.6	58.6
31	31.0	53.0	56.4
32	28.4	48.9	49.7
33	30.5	38.7	67.5
34	28.2	40.7	64.4
35	26.1	41.2	41.9
36	30.2	44.9	54.2
37	35.6	45.9	72.0
38	29.9	42.9	59.5

39	31.5	54.9	48.3
40	34.2	54.6	65.9
41	56.8	92.0	97.6
42	23.5	91.4	42.2
43	21.9	54.0	39.0
44	20.5	37.7	55.6
45	25.4	42.5	59.5
46	23.9	47.4	37.9
47	19.9	35.6	38.6
48	21.1	34.9	46.7
49	18.7	35.2	34.8
50	19.7	38.7	29.1
51	28.9	47.0	42.7
52	23.1	52.8	39.9
53	15.9	34.7	40.8
54	13.2	29.1	26.1

Table S6. ^1H-^{15}N RDCs of 6HB obtained in a positively charged stretched polyacrylamide gel, measured at 800 MHz and 35 °C. Couplings listed ignore the negative sign of the ^{15}N gyromagnetic ratio.

Residue	$^1D_{NH}$ (Hz)	Error (Hz)
7	-17.2	0.6
8	-19.7	0.9
9	-17.4	0.8
10	-16.3	1.5
11	-18.2	0.7
12	-18.3	1.9
14	-15.9	0.9
15	-16.3	1.4
16	-17.7	0.7
17	-17.5	1.2
18	-14.9	1.3
19	-18.3	1.1
20	-19.0	0.9
21	-12.7	1.4
22	-17.8	0.9
23	-20.5	1.1
24	-14.1	0.9
25	-16.8	1.0
26	-18.6	0.9
27	-17.5	0.5
28	-16.5	0.6
29	-17.5	0.6
30	-19.3	0.8
31	-14.8	0.8
32	-15.8	0.9
33	-18.7	0.9
34	-18.4	0.8
35	-14.5	0.6
36	-15.0	0.7
37	-18.8	0.7
38	-16.2	0.7
39	-12.0	0.5
40	-18.5	0.4
41	-16.0	0.8

42	-13.8	0.5
43	-15.0	0.6
44	-18.0	0.6
45	-16.2	0.5
46	-12.7	0.7
47	-17.2	0.6
48	-18.4	0.6
49	-15.4	0.5
50	-13.5	0.5
51	-13.0	0.5
52	-3.8	0.4
53	-2.8	0.2
54	2.3	0.2
56	-4.1	0.2
57	0.2	1.5
58	5.0	1.4
59	-1.5	0.4
60	1.1	0.2
61	-3.0	0.1
62	5.0	0.2
63	0.4	0.3
64	9.7	0.2
65	9.4	0.2
66	9.6	0.4
67	9.7	0.2
69	-14.4	0.7
70	-10.5	0.4
71	-15.4	0.7
72	-15.7	0.6
74	-10.7	0.6
75	-17.8	0.9
76	-13.4	0.8
77	-9.1	0.6
78	-14.8	0.8
79	-18.8	0.8
80	-11.0	0.7
81	-11.9	1.2
82	-16.9	1.4
83	-12.4	0.6

84	-13.9	0.7
85	-12.4	0.9
86	-0.2	0.5
87	7.0	1.7
88	9.4	0.5
89	-7.9	0.4
90	-12.9	0.3
91	-12.9	0.3
92	-6.8	0.3
93	-3.7	0.5
94	-9.6	0.2
95	-5.4	0.2
96	1.3	0.1
